# LIN28B promotes cell invasion and colorectal cancer metastasis via CLDN1 and NOTCH3

**DOI:** 10.1172/jci.insight.167310

**Published:** 2023-07-24

**Authors:** Kensuke Sugiura, Yasunori Masuike, Kensuke Suzuki, Alice E. Shin, Nozomu Sakai, Hisahiro Matsubara, Masayuki Otsuka, Peter A. Sims, Christopher J. Lengner, Anil K. Rustgi

**Affiliations:** 1 Herbert Irving Comprehensive Cancer Center, Division of Digestive and Liver Diseases, Department of Medicine, Vagelos College of Physicians and Surgeons, Columbia University Irving Medical Center, New York, New York, USA.; 2 Department of General Surgery and; 3 Department of Frontier Surgery, Graduate School of Medicine, Chiba University, Chiba, Japan.; 4 Department of Systems Biology and Department of Biochemistry & Molecular Biophysics, Herbert Irving Comprehensive Cancer Center, Vagelos College of Physicians and Surgeons, Columbia University Irving Medical Center, New York, New York, USA.; 5 Department of Biomedical Sciences, School of Veterinary Medicine, University of Pennsylvania, Philadelphia, Pennsylvania, USA.

**Keywords:** Gastroenterology, Oncology, Cancer, Colorectal cancer

## Abstract

The RNA-binding protein LIN28B is overexpressed in over 30% of patients with colorectal cancer (CRC) and is associated with poor prognosis. In the present study, we unraveled a potentially novel mechanism by which LIN28B regulates colonic epithelial cell-cell junctions and CRC metastasis. Using human CRC cells (DLD-1, Caco-2, and LoVo) with either knockdown or overexpression of LIN28B, we identified claudin 1 (CLDN1) tight junction protein as a direct downstream target and effector of LIN28B. RNA immunoprecipitation revealed that LIN28B directly binds to and posttranscriptionally regulates *CLDN1* mRNA. Furthermore, using in vitro assays and a potentially novel murine model of metastatic CRC, we show that LIN28B-mediated *CLDN1* expression enhances collective invasion, cell migration, and metastatic liver tumor formation. Bulk RNA sequencing of the metastatic liver tumors identified NOTCH3 as a downstream effector of the LIN28B/CLDN1 axis. Additionally, genetic and pharmacologic manipulation of NOTCH3 signaling revealed that NOTCH3 was necessary for invasion and metastatic liver tumor formation. In summary, our results suggest that LIN28B promotes invasion and liver metastasis of CRC by posttranscriptionally regulating CLDN1 and activating NOTCH3 signaling. This discovery offers a promising new therapeutic option for metastatic CRC to the liver, an area where therapeutic advancements have been relatively scarce.

## Graphical abstract

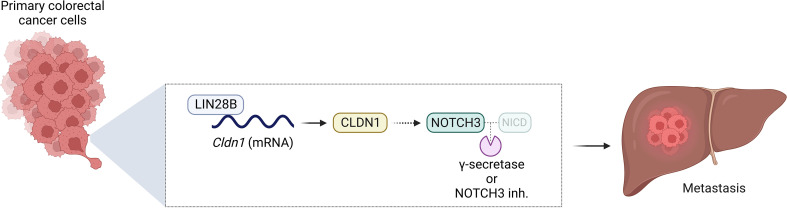

## Introduction

Colorectal cancer (CRC) is the third most commonly diagnosed cancer and the second most common cause of cancer death in the world (1). The 5-year survival rate in patients with localized tumors is 90%, but the survival rate drastically decreases to 14% in patients with distant metastasis despite various interventions such as chemotherapy, immunotherapy, and surgery (2, 3). Therefore, it is imperative to elucidate the molecular mechanisms underlying CRC metastasis, especially to the liver, a common site for colonization and outgrowth.

LIN28 is an evolutionarily conserved RNA-binding protein (RBP), initially identified in *Caenorhabditis elegans* as having developmental functions (4). In mammals, there are 2 paralogs of LIN28, LIN28A and LIN28B, both sharing similar domain structure and function. LIN28A plays a key role in maintaining embryonic stem cell pluripotency and enhances epigenetic reprogramming by OCT4/SOX2/KLF4 (5). LIN28B has also been suggested to play a role in pluripotency (6); however, its role in cancer has been more extensively investigated due to increasing evidence demonstrating that LIN28B serves as an oncogene. For example, LIN28B is associated with advanced stage and early recurrence of hepatocellular carcinoma (7). In esophageal cancer, LIN28B overexpression promotes cell invasion in vitro and correlates with poor overall and disease-free survival (8). In CRC, LIN28B is overexpressed in 30%–60% of patients and associated with poor prognosis (9–11). Additionally, we previously reported that LIN28B acts as an oncogene in a genetic mouse model of CRC and promotes liver metastasis in a subcutaneous xenograft model (9, 12). Thus, LIN28B is associated with poor prognosis and tumor metastasis in diverse cancers. However, the underlying molecular mechanisms of how LIN28B functions in tumor metastasis, especially in CRC, are unclear and require elucidation for potential translational therapeutics.

*Let-7* microRNA (miRNA) is downregulated in various human cancers. LIN28 directly targets the *let-7* family, inhibiting its biogenesis. Similar to that of LIN28B, *let-7* dysregulation is associated with poor prognosis and increased metastatic potential in several cancer types, including breast, lung, colorectal, and ovarian cancers (13). Although many studies have investigated the role of the LIN28B and *let-7* loop in promoting cancer hallmarks, such as cell proliferation, metabolism, evasion of immune destruction, and metastasis (13), other potential targets of LIN28B and their contribution to cancer progression have received less attention.

We previously performed cross-linking immunoprecipitation sequencing (3-Seq; CLIP-Seq) analysis of the target mRNAs of LIN28B and revealed that LIN28B binds to various adherens and tight junction mRNAs, including claudin 1 (*CLDN1*) (12). Among the 27 claudin family members discovered to date, CLDN1 is strongly implicated in cancer progression (14–16). For example, CLDN1 promotes invasiveness in CRC and hepatocellular carcinoma (17, 18). Additionally, CLDN1 regulates growth and metastasis in mouse models of CRC (19). The invasive function of CLDN1 may be a consequence of it being a tight junction protein and its integral role in maintaining cell-cell contacts. Cell-cell interaction is required for an important step in cancer metastasis called collective cell invasion, a type of cell migration that retains cell-cell contacts to form a structural and functional unit (20–23). Therefore, we hypothesized that LIN28B binds to and promotes translation of *CLDN1*, resulting in collective cell invasion and subsequent metastasis of CRC.

The Notch signaling pathway regulates cell proliferation, differentiation, and maturation in several tissues and cell types. Notch signals are activated and transduced when NOTCH1–4 transmembrane receptors bind to transmembrane ligands expressed on adjacent cells. Activation of the Notch receptors initiates a proteolytic cascade via γ-secretase, leading to the transcription of downstream target genes, including *HES* and *HEY* transcription factors. The targets of these transcription factors regulate cell differentiation, maturation, and proliferation (24). As such, dysregulated Notch signaling has also been implicated in several human cancers. For example, NOTCH3 overexpression is associated with poorly differentiated colorectal tumors (25), increased tumor growth rate (26), higher rates of venous invasion, and shorter recurrence-free survival (25).

In the present study, we investigate the role of LIN28B in regulating cell-cell junctions and the functional consequences of modulating LIN28B expression on CRC progression. We show that LIN28B promotes collective cell invasion through posttranscriptional upregulation of *CLDN1* gene expression. Furthermore, we demonstrate that LIN28B-mediated upregulation of CLDN1 induces NOTCH3 signaling to promote liver metastasis of CRC, and genetic modulation as well as pharmacologic inhibition of NOTCH signaling reduce metastasis. Our results suggest that a delineated LIN28B/CLDN1/NOTCH3 axis regulates CRC metastasis to the liver, thereby providing new perspectives on therapy of this deadly manifestation of CRC.

## Results

### LIN28B promotes cell migration and collective cell invasion of CRC cells.

To study the functional role of LIN28B in CRC, we generated CRC cells with genetic modification of LIN28B expression. Endogenous LIN28B expression is high in human Caco-2 CRC cells, whereas it is low in human DLD-1 cells and LoVo CRC cells (27, 28). Thus, we generated Caco-2 cells with LIN28B knockdown by using short hairpin RNA (shRNA) and DLD-1 and LoVo cells with LIN28B overexpression (LIN28B^hi^). The downregulation and upregulation of LIN28B protein levels were verified by immunoblotting (Supplemental Figure 1, A and B; supplemental material available online with this article; https://doi.org/10.1172/jci.insight.167310DS1).

**Figure 1:**
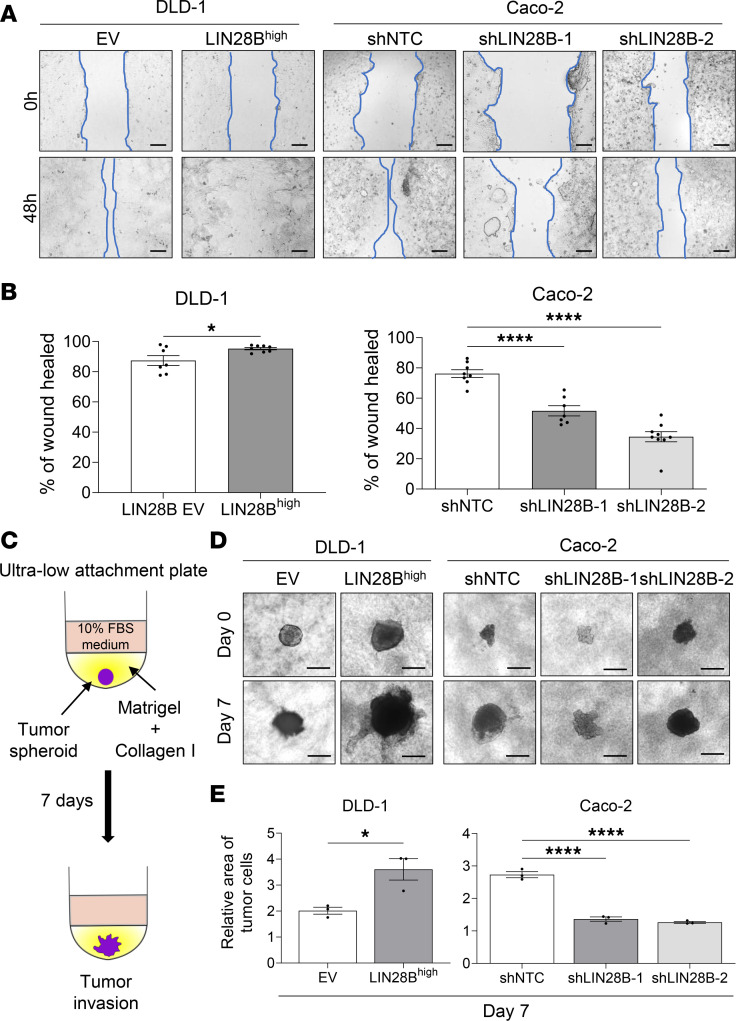
LIN28B promotes cell migration and collective cell invasion in colon cancer cells. (**A**) A comparison of wound closure in DLD-1 cells with an empty vector versus LIN28B overexpression (left) and in Caco-2 cells with shControl versus shLIN28B for LIN28B knockdown. Scale bar represents 200 μm. (**B**) The wound area in **A** is quantified using ImageJ (NIH). The wound-healing rate is defined as (initial wound area – wound area at 24 or 48 hours)/initial wound area. Data were analyzed using a 2-tailed Student’s *t* test or 1-way ANOVA and are represented as mean ± SEM (*n* = 7). (**C**) A schematic model of the tumor spheroid 3D invasion assay. (**D**) 3D invasion of DLD-1 cells with an empty vector versus LIN28B overexpression and of Caco-2 cells with shControl versus shLIN28B is shown at day 0 and day 7. Scale bar = 500 μm. (**E**) The area of the tumor cells in **D** is quantified using ImageJ. Data were analyzed using a 2-tailed Student’s *t* test or 1-way ANOVA, expressed relative to the corresponding value at day 0, and are represented as mean ± SEM (*n* = 3). *P < 0.05, ****P < 0.0001.

Next, to investigate whether LIN28B mediates cell invasion and migration, we compared the wound-healing capacities of CRC cells with LIN28B-high versus -low expression. DLD-1 LIN28B^hi^, Caco-2 control (Ctrl) (with endogenous high LIN28B level), and LoVo LIN28B^hi^ cells had significantly higher wound-healing rates at 48 hours after the initial scratch (Figure 1, A and B, and Supplemental Figure 1B). Transwell 2D migration and invasion assays also revealed that DLD-1 LIN28B^hi^ and LoVo LIN28B^hi^ were significantly more migratory and invasive (Supplemental Figure 1, C and D). Of note, the Transwell assays did not work with the Caco-2 cells, perhaps reflecting their intrinsic well-differentiated status (data not shown). The 3D ECM-based assay is used commonly to assess collective cell invasion in vitro (29, 30). Therefore, we performed spheroid 3D invasion assay using ultra-low-attachment plates (Figure 1C). By day 7, DLD-1 LIN28B^hi^, Caco-2 Ctrl, and LoVo LIN28B^hi^ cells invaded to a greater area when compared with LIN28B^lo^-expressing cells (Figure 1, D and E, and Supplemental Figure 1E). These results suggest that LIN28B enhances migration and collective cell invasion of CRC cells.

### LIN28B enhances CLDN1 expression and cell aggregation of CRC cells.

**Figure 2:**
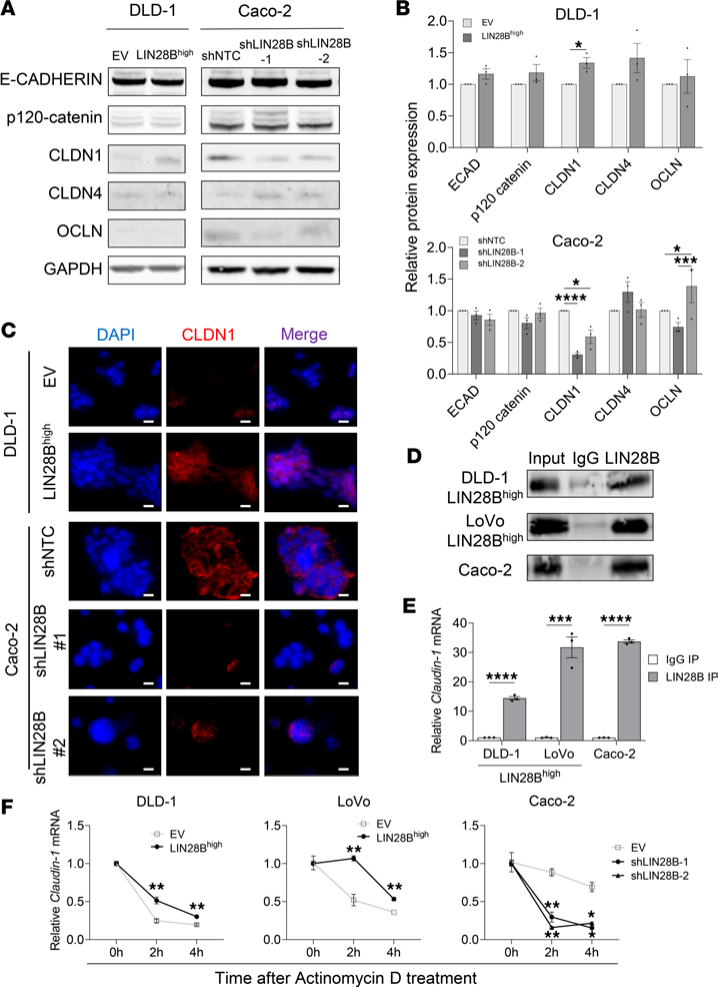
LIN28B directly binds to and stabilizes *CLDN1* mRNA. (**A**) Western blot analysis of adherens and tight junctions from DLD-1 and Caco-2 cells. (**B**) Quantification of band densities measured by Western blot in **A**, normalized to GAPDH. Data were analyzed using a 2-tailed Student’s *t* test or 1-way ANOVA, expressed relative to the corresponding value in empty vector or control groups, and represented as means ± SEM (*n* = 3). (**C**) Immunofluorescence staining of CLDN1 (shown in red) in DLD-1 and Caco-2 cells. Nuclei were stained by DAPI (shown in blue). Scale bar = 100 μm. (**D**) Representative images of Western blots of samples precipitated using IgG or anti-LIN28B antibodies. (**E**) qRT-PCR analysis of *claudin-1* mRNA in RNA immunoprecipitation samples. Data were analyzed using a 2-tailed Student’s *t* test, expressed relative to the corresponding value of IgG IP samples, and represented as means ± SEM (*n* = 3). (**F**) Quantification of *claudin-1* mRNA after actinomycin D treatment. Data were analyzed using a 2-way ANOVA, expressed relative to the corresponding value at 0 hour, and represented as means ± SEM (*n* = 3). *P < 0.05, **P < 0.01, ***P < 0.001, ****P < 0.0001. qRT-PCR, quantitative real-time PCR.

We have previously performed CLIP-Seq, which revealed various adherens and tight junction RNA transcripts as targets of LIN28B (12). To follow up with these findings, we measured the protein expression levels of the following CLIP-Seq target transcripts: E-CADHERIN and p120-catenin as representatives of the adherens junctions and CLDN1, CLDN4, and occludin (OCLN) as representatives of the tight junctions. DLD-1 cells and LoVo cells with LIN28B overexpression (LIN28B^hi^) had significantly higher CLDN1 protein levels than the corresponding empty vector (EV) controls (Figure 2, A and B, and Supplemental Figure 2, A and B). Similarly, Caco-2 cells with LIN28B knockdown had significantly lower CLDN1 protein levels than the control cells (Caco-2 Ctrl) (Figure 2, A and B, and Supplemental Figure 2, A and B). By contrast, E-CADHERIN, p120-catenin, CLDN4, or OCLN protein levels were not significantly changed between LIN28B^lo^ and LIN28B^hi^ CRC cells (Figure 2, A and B, and Supplemental Figure 2, A and B). Immunofluorescence of CLDN1 protein expression further validated higher CLDN1 expression in DLD-1 LIN28B^hi^, Caco-2 Ctrl, and LoVo LIN28B^hi^ cells when compared with their respective control groups (Figure 2C and Supplemental Figure 2C).

Due to the pivotal role of CLDN1 as a tight junction protein and the revealed association between LIN28B and CLDN1 transcript, we hypothesized that LIN28B expression enhances cellular aggregation. To evaluate this, we measured cellular aggregation of LIN28B expression–modified CRC cells in ultra-low-attachment plates. This revealed that DLD-1 LIN28B^hi^, Caco-2 Ctrl, and LoVo LIN28B^hi^ cells promoted greater cell aggregation when compared with DLD-1 Ctrl, Caco-2 LIN28B^lo^, and LoVo Ctrl cells, respectively (Supplemental Figure 3, A and B). These findings support the notion that LIN28B enhances CLDN1 expression and cell aggregation of CRC cells.

### LIN28B directly binds to and stabilizes CLDN1 mRNA.

We next focused on identifying the mechanism(s) by which LIN28B regulates CLDN1 expression in CRC cells. As an RBP, LIN28B is responsible for posttranscriptional regulation, such as RNA splicing, transport, stability, and localization (31–33). Therefore, we hypothesized that LIN28B binds to *CLDN1* mRNA and regulates it in a posttranscriptional fashion. We first used an anti-LIN28B antibody to perform RNA immunoprecipitation (RIP) in DLD-1 LIN28B^hi^, LoVo LIN28B^hi^, and Caco-2 cells. Western blot of the precipitated samples verified the efficiency of the anti-LIN28B antibody (Figure 2D). The amount of *CLDN1* mRNA precipitate was significantly greater than the amount of *CLDN1* mRNA pulled down by the IgG negative control antibody in LIN28B^hi^ cells (Figure 2E).

To further clarify how LIN28B regulates CLDN1 protein expression, *CLDN1* mRNA stability was evaluated by using actinomycin D, a transcription inhibitor that intercalates into DNA to prevent RNA polymerase activity (34–36). Upon treatment of CRC cells with actinomycin D, *CLDN1* mRNA decay was analyzed every 2 hours by quantitative real-time PCR (qRT-PCR). Our quantification revealed delayed decay of *CLDN1* mRNA expression in LIN28B^hi^ cells after actinomycin D treatment (Figure 2F), supporting our hypothesis that LIN28B upregulates CLDN1 protein expression by binding to and stabilizing *CLDN1* mRNA.

### LIN28B-induced cell aggregation and collective cell invasion are dependent on CLDN1.

**Figure 3:**
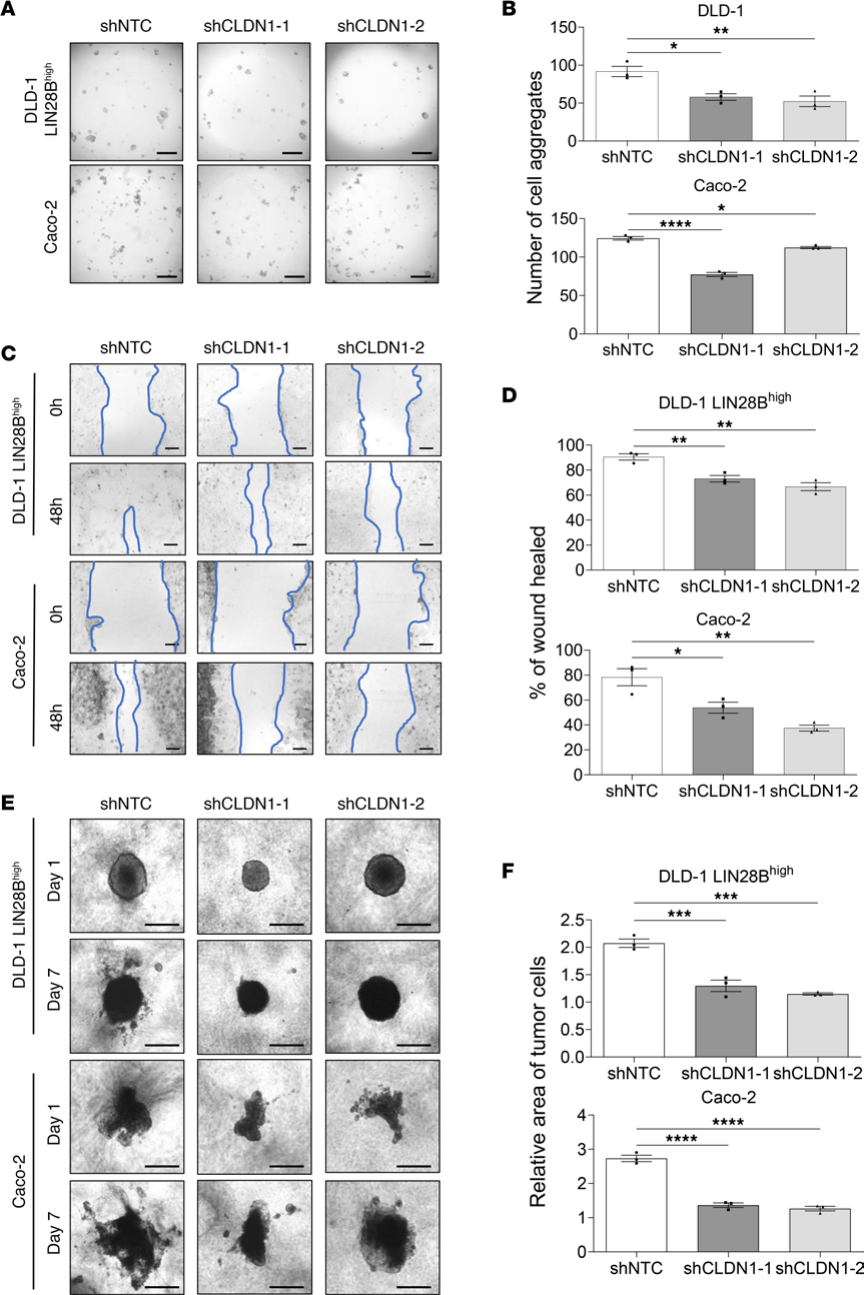
LIN28B-induced cell aggregation and collective cell invasion are dependent on CLDN1. (**A**) Representative images of cell aggregates formed from DLD-1 LIN28B^hi^ and Caco-2 cells with CLDN1 knockdown after a 24-hour incubation in ultra-low-attachment plates. Scale bar = 500 μm. (**B**) Quantification of the number of cell aggregates by a Keyence BZ-X810 microscope. (**C**) Representative images of wound closure of DLD-1 LIN28B^hi^ and Caco-2 cells with CLDN1 knockdown. Scale bar = 200 μm. (**D**) The wound-healing rate in **C** was analyzed using ImageJ. Data are represented as means ± SEM (*n* = 3). (**E**) 3D invasion of DLD-1 LIN28B^hi^ and Caco-2 cells with CLDN1 knockdown at 1 and 7 days after incubation. Scale bar = 500 μm. (**F**) Tumor area in **E** was quantified by ImageJ. Data are expressed relative to the corresponding value at day 1 and represented as means ± SEM. All graphs were generated from data analyzed by 1-way ANOVA followed by Tukey’s multiple-comparison test. *P < 0.05, **P < 0.01, ***P < 0.001, ****P < 0.0001.

To determine whether CLDN1 upregulation plays a pivotal role in collective cell invasion induced by LIN28B, we suppressed CLDN1 expression in DLD-1 LIN28B^hi^, LoVo LIN28B^hi^, and Caco-2 cells by 2 shRNAs. We verified first that CLDN1 mRNA and protein expression was significantly suppressed in shCLDN1 cells of all 3 cell lines (Supplemental Figure 4, A and B). Next, to verify whether CLDN1 knockdown inhibits LIN28B-induced cell aggregation, we compared the ability of shCLDN1 and control cells to aggregate. The number of cell aggregates was significantly lower in shCLDN1 cells (Figure 3, A and B, and Supplemental Figure 4C). We performed a wound-healing assay to examine the role of CLDN1 in LIN28B-induced cell invasion and migration. shCLDN1 cells displayed a significantly lower wound-healing rate, suggesting reduced migratory abilities in CLDN1-knockdown cells (Figure 3, C and D, and Supplemental Figure 4D). Moreover, the 3D tumor spheroid invasion assay confirmed that shCLDN1 cells invaded to a smaller area than control cells (Figure 3, E and F, and Supplemental Figure 4E). Taken together, these results indicate that CLDN1 contributes to LIN28B-induced cell aggregation and collective cell invasion.

### LIN28B-induced CLDN1 upregulation promotes metastatic liver tumor formation.

**Figure 4:**
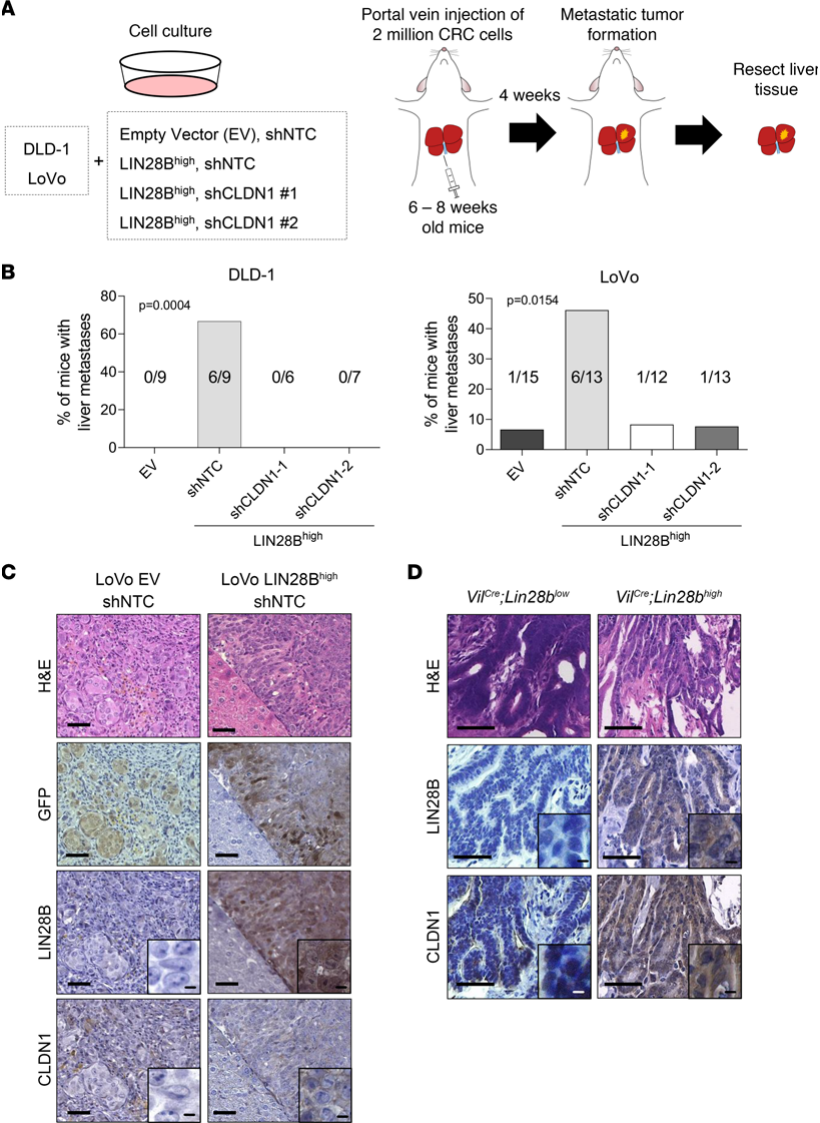
LIN28B-induced CLDN1 upregulation promotes metastatic liver tumor formation. (**A**) Schematic drawing of the workflow used to study the effects of CLDN1 knockdown in liver metastasis formation in vivo. (**B**) Quantification of the proportion of mice with metastatic liver tumors (*n* ≥ 6), where left graph depicts results from injection of DLD-1 cells, and right graph depicts results from injection of LoVo cells. Statistical analyses were performed using χ^2^ test. (**C**) Representative images of H&E and IHC staining for GFP, LIN28B, and CLDN1 in metastatic liver tumors from LoVo with empty vector and shNTC group (left) and LoVo with LIN28B overexpression and shNTC group (right). Scale bar = 50 μm. (**D**) Representative images of H&E and IHC staining for LIN28B and CLDN1 in intestinal tumors from a *Villin^Cre^ Lin28b^lo^* mouse (left, *n* = 1) and *Villin^Cre^ Lin28b^hi^* mice (right, *n* = 3). Scale bar = 50 μm. Scale bar for insets = 200 μm.

Portal vein injection of LIN28B^hi^ CRC cells results in liver metastasis formation by undergoing extravasation, colonization, and outgrowth (37). Given this, we next explored the potential role of CLDN1 in LIN28B-induced liver metastasis. LIN28B^hi^ CRC cells with GFP fluorescence were injected into the portal vein of 6- to 8-week-old Taconic NCr nude mice (*CrTac NCr-Foxn1^nu^*), and the liver tissues were harvested 4 weeks after injection (Figure 4A). Experiments using Caco-2 cells were excluded because previous studies showed that the cell line does not form liver metastases in mice (37–39), potentially due to higher expression of *let-7* miRNA in Caco-2 cells (Supplemental Figure 4F). As expected, injection of parental DLD-1 cells (EV) with nontarget control shRNA (shNTC) did not result in liver tumors (0/9, 0%), and injection of DLD-1 LIN28B^hi^ cells with shNTC induced liver metastases (6/9, 66.7%) (Figure 4B). Similarly, LoVo LIN28B^hi^ cells with shNTC formed more liver metastases (6/13, 46.2%) than LoVo EV with shNTC (1/15, 6.7%) (Figure 4B). Intriguingly, knockdown of CLDN1 completely inhibited liver tumor formation in mice injected with DLD-1 LIN28B^hi^ cells (0/6 and 0/7, 0%) (Figure 4B). Similarly, the number of liver tumors in mice injected with LoVo LIN28B^hi^ was decreased upon CLDN1 knockdown (Figure 4B). The presence of metastatic liver tumors was verified by GFP staining (Figure 4C). Histological analysis showed that tumors derived from LoVo LIN28B^hi^ cells had higher expression of CLDN1 than tumors with EV, consistent with our in vitro experiments (Figure 4C). We have previously published that overexpression of LIN28B in intestinal epithelial cells (*Vil^Cre^ Lin28b^hi^*) induces spontaneous tumorigenesis (without liver metastasis) in a transgenic mouse model (12). Analysis of intestinal tumors from *Vil^Cre^ Lin28b^hi^* mice showed that the tumors with higher LIN28B expression had higher CLDN1 expression when compared with tumors from *Vil^Cre^ Lin28b^lo^* mice (Figure 4D).

### NOTCH3 regulates collective invasion and liver metastasis formation downstream of LIN28B/CLDN1 axis.

To identify downstream targets of LIN28B/CLDN1 axis, we performed bulk RNA sequencing (RNA-Seq) of the liver tumors generated from portal vein injection of DLD-1 LIN28B^hi^ and LoVo LIN28B^hi^ cells with or without CLDN1 depletion. Among commonly downregulated genes upon CLDN1 depletion, we selected NOTCH3 as a potential downstream regulator of CLDN1 (Supplemental Figure 5). We reasoned that cell-cell junctions (e.g., CLDN1 as a component of tight junctions) are required for the ligand-receptor interaction to induce NOTCH-mediated signaling. Additionally, there is evidence that CLDN1 regulates intestinal homeostasis through NOTCH signaling (40). Interestingly, NOTCH3 oncogenic signaling has been implicated in invasiveness and metastasis of various cancer types (41, 42). Therefore, we hypothesized that NOTCH3 acts downstream of LIN28B/CLDN1 axis to regulate collective invasion and subsequent liver metastasis formation.

**Figure 5:**
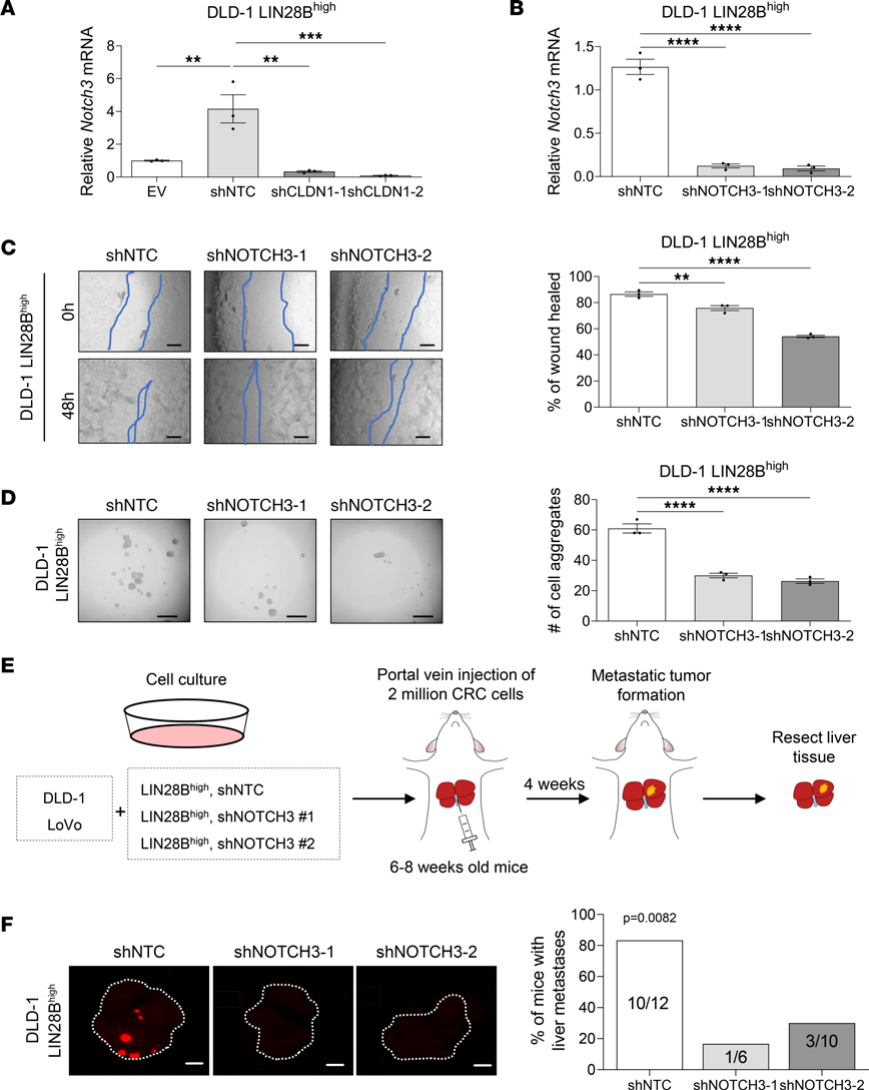
NOTCH3 regulates collective cell invasion and liver metastasis formation downstream of LIN28B/CLDN1 axis. (**A**) qRT-PCR analysis of *NOTCH3* mRNA in DLD-1 LIN28B^hi^ cells with or without shCLDN1. (**B**) qRT-PCR analysis of *NOTCH3* mRNA in DLD-1 LIN28B^hi^ cells with or without shNOTCH3. (**C**) Wound closure of DLD-1 LIN28B^hi^ cells with or without shNOTCH3. Scale bar = 200 μm (left). Wound-healing rates were quantified by ImageJ (right graph). Data represented as means ± SEM (*n* = 3). (**D**) Representative images of aggregates formed from DLD-1 LIN28B^hi^ cells transduced by shNOTCH3 (left). The number of cell aggregates was counted by Keyence BZ-X810 (right graph). Data represented as means ± SEM (*n* = 3). (**E**) Schematic drawing of the workflow used to study the effects of NOTCH3 knockdown on portal vein injection model of metastatic CRC. (**F**) Representative images of RFP expressed by DLD-1 LIN28B^hi^ tumors in the liver. Images were taken using Keyence BZ-X810. Scale bar = 500 μm. Data in **A**–**D** were analyzed by 1-way ANOVA followed by Tukey’s multiple-comparison test. Data in **F** were analyzed by χ^2^ test. **P < 0.01, ***P < 0.001, ****P < 0.0001.

To determine whether NOTCH3 regulates LIN28B-mediated effects in vitro, we suppressed *NOTCH3* expression in LoVo and DLD-1 LIN28B^hi^ cells. We first verified downregulation of *NOTCH3* in LIN28B^hi^ shNOTCH3 and LIN28B^hi^ shCLDN1 cells by qRT-PCR (Figure 5, A and B, and Supplemental Figure 6, A and B). Of note, we also measured *CLDN1* mRNA expression in DLD-1 and LoVo LIN28B^hi^ shNOTCH3 cells and observed no significant difference between the groups, verifying that NOTCH3 acts downstream of CLDN1 (Supplemental Figure 6C). We next performed wound-healing and cell aggregation assays to assess the invasion, migration, and aggregation abilities of LIN28B^hi^ shNOTCH3 CRC cells. When analyzed 48 hours after the scratch, DLD-1 LIN28B^hi^ and LoVo LIN28B^hi^ cells had significantly higher wound-healing rates compared with LIN28B^hi^ NOTCH3-knockdown cells (Figure 5C and Supplemental Figure 6D). Similarly, quantification of cell aggregation, colony formation, and spheroid formation between LIN28B^hi^ shNOTCH3 and LIN28B^hi^ shRNA Ctrl cells showed that the number of cell aggregates, colonies, and spheres was significantly lower in NOTCH3-knockdown cells (Figure 5D, Supplemental Figure 6E, and Supplemental Figure 9). These results underscore that NOTCH3 contributes to LIN28B-induced cell aggregation and collective cell invasion.

To validate whether NOTCH3 mediates liver metastasis formation downstream of LIN28B in vivo, LIN28B^hi^ shNOTCH3 cells labeled with RFP were injected into the portal vein of immunocompromised mice, and the liver tissues were harvested 4 weeks after injection (Figure 5E). As expected, injection of LIN28B^hi^ cells with shNTC readily formed liver metastases in both DLD-1 (10/12, 83.3%) and LoVo (7/9, 77.8%) cells (Figure 5F and Supplemental Figure 6F). Importantly, injection of LIN28B^hi^ shNOTCH3 cells significantly reduced the formation of liver metastases in both DLD-1 cells (1/6, 16.7% and 3/10, 30%) and LoVo cells (1/6, 16.7% and 2/7, 28.6%) (Figure 5F and Supplemental Figure 6F).

### Pharmacologic inhibition of Notch signaling pathway reduces LIN28B-induced liver metastasis.

**Figure 6:**
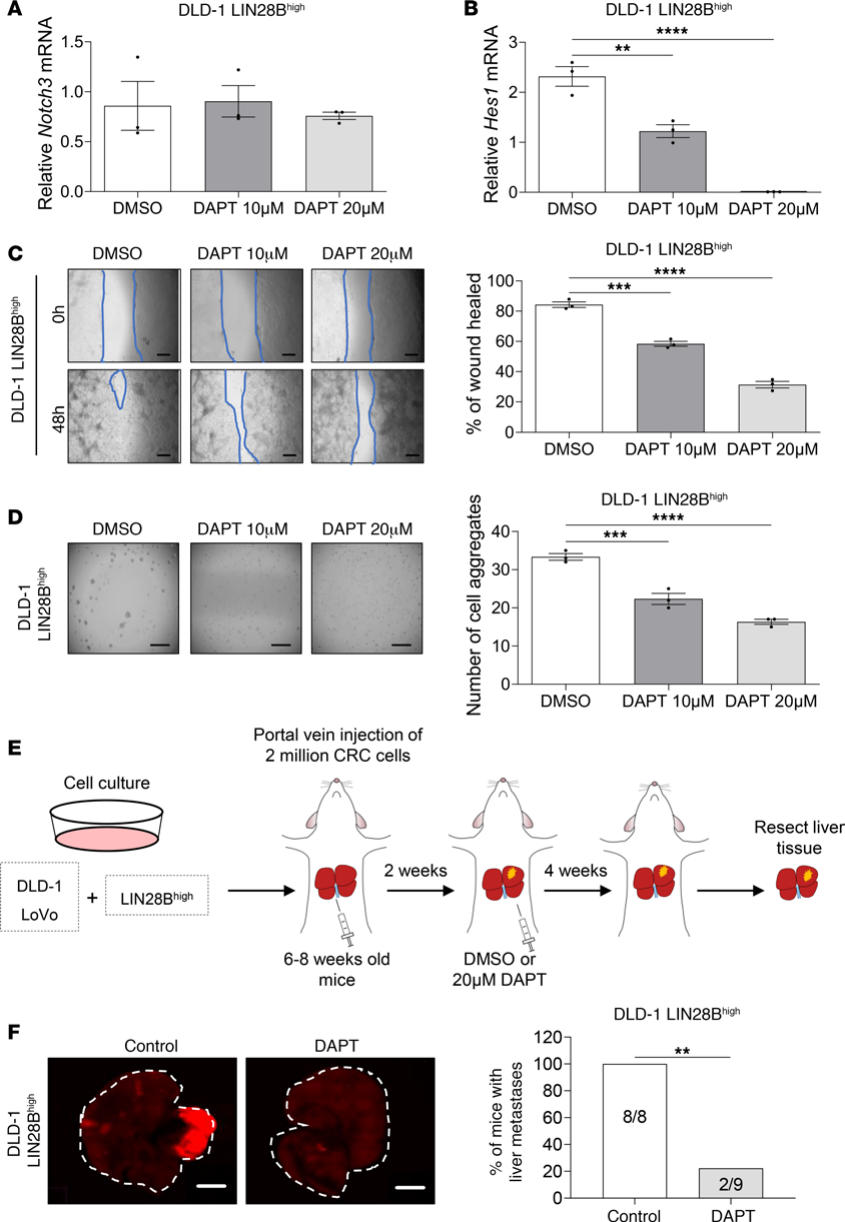
Pharmacologic inhibition of Notch signaling reduces LIN28B-induced liver metastasis. (**A**) qRT-PCR analysis of *NOTCH3* mRNA in DLD-1 LIN28B^hi^ cells treated with DMSO, 10 μM DAPT, or 20 μM DAPT. (**B**) qRT-PCR analysis of *HES1* mRNA in DLD-1 LIN28B^hi^ cells treated with DMSO, 10 μM DAPT, or 20 μM DAPT. (**C**) Representative images of the wound closure scratch assay performed using DLD-1 LIN28B^hi^ cells treated with DMSO, 10 μM DAPT, or 20 μM DAPT. Scale bar = 200 μm. Area of the wound was measured by using ImageJ. Data represented as means ± SEM (*n* = 3). (**D**) Representative images of the aggregation assay performed using DLD-1 LIN28B^hi^ cells treated with DMSO, 10 μM DAPT, or 20 μM DAPT. The number of cell aggregates was counted using Keyence BZ-X810. Data represented as means ± SEM (*n* = 3). (**E**) Schematic drawing of the workflow used to study the effects of DAPT injection in a portal vein injection model of CRC metastasis. (**F**) Representative images of RFP expressed by DLD-1 LIN28B^hi^ tumors in the liver. Images were taken using Keyence BZ-X810. Scale bar = 500 μm. Data in **A**–**D** were analyzed by 1-way ANOVA followed by Tukey’s multiple-comparison test. Data in **F** were analyzed by Fisher’s exact test. **P < 0.01, ***P < 0.001, ****P < 0.0001.

To evaluate the potential of Notch signaling as a therapeutic target, we used a γ-secretase inhibitor, DAPT, to study the effects of Notch signaling inhibition in vivo and in vitro. qRT-PCR analysis showed no significant difference in *NOTCH3* mRNA expression between DAPT-treated and DMSO vehicle–treated LIN28B^hi^ CRC cells (Figure 6A and Supplemental Figure 7A). However, DAPT treatment resulted in a significant decrease in the expression of hairy and enhancer of split homolog-1 (*HES1*), a downstream effector target for mammalian Notch signaling pathway (21) (Figure 6B and Supplemental Figure 7B).

We next compared the effects of DAPT on the invasiveness of LIN28B^hi^ CRC cells. DAPT treatment significantly reduced wound-healing rates at 48 hours after the initial scratch (Figure 6C and Supplemental Figure 7C), suggesting that pharmacologic inhibition of Notch signaling reduces migration and invasion of CRC cells. Similarly, comparison of cell aggregation between DAPT treatment group and the vehicle-treated control group revealed significantly reduced number of cell aggregates upon DAPT treatment (Figure 6D and Supplemental Figure 7D).

To evaluate the effects of DAPT treatment on the formation of liver metastasis in vivo, LIN28B^hi^ CRC cells labeled with mCherry fluorescence were injected into the portal vein of immunocompromised mice. Two weeks after portal vein injection, the mice received intraperitoneal injection of DMSO or DAPT 3 times per week for 4 weeks. At the experimental endpoint, liver tissues were harvested for analyses (Figure 6E). All mice injected with DLD-1 LIN28B^hi^ cells and treated with DMSO developed liver metastases as visualized by RFP fluorescence (8/8, 100%), whereas mice treated with DAPT developed significantly fewer liver tumors (2/9, 22.2%) (Figure 6F). Similarly, mice injected with LoVo LIN28B^hi^ cells and treated with DMSO formed more liver metastases (4/7, 57.1%) than the DAPT-treated experimental group (0/8, 0%) (Supplemental Figure 7E). These results support the potentially novel finding that LIN28B/CLDN1/NOTCH3 axis contributes to cell aggregation and collective cell invasion, and Notch signaling pathway might be a promising pharmacologic target for the treatment of metastatic colon cancer.

### Pharmacologic inhibition of NOTCH3 reduces LIN28B-induced liver metastasis.

**Figure 7:**
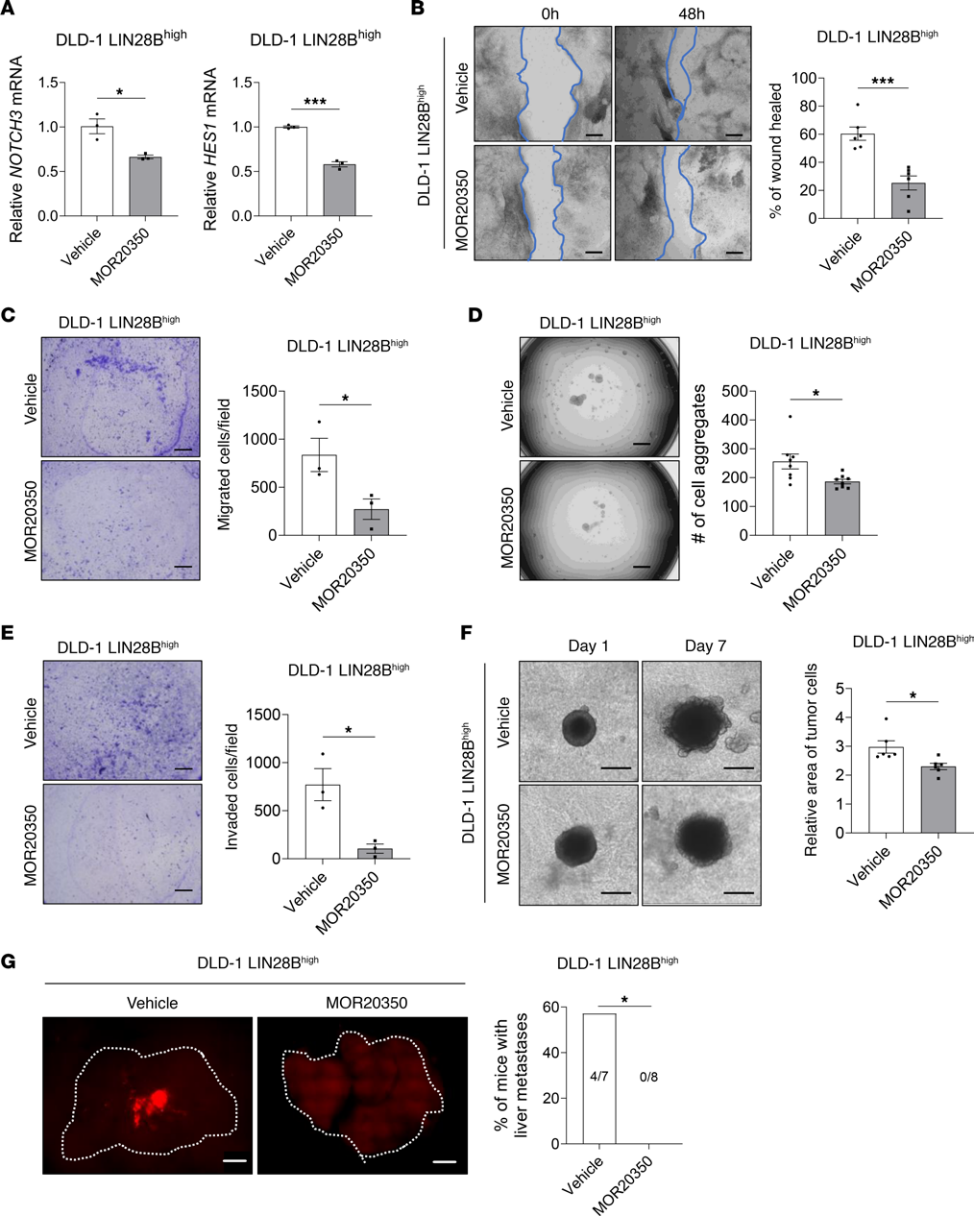
Pharmacologic inhibition of NOTCH3 reduces LIN28B-induced liver metastasis. (**A**) qRT-PCR analysis of *NOTCH3* and *HES1* mRNA in DLD-1 LIN28B^hi^ cells treated with vehicle or 10 μg/mL MOR20350 for 48 hours. (**B**) Representative images and quantification of the wound closure scratch assay performed using DLD-1 LIN28B^hi^ cells treated with vehicle or MOR20350. Scale bar = 200 μm. Area of the wound was measured by using ImageJ. (**C**) Representative images and quantification of the 3D aggregation assay performed using DLD-1 LIN28B^hi^ cells treated with the vehicle or MOR20350. The number of cell aggregates was counted using Keyence BZ-X810. (**D**) Representative images and quantification of 2D invasion assay. Cells that have invaded through the 8 μm pore and the ECM were counted using Keyence BZ-X810. (**E**) Representative images and quantification of 2D migration assay. Cells that have migrated through the 8 μm pore were counted using Keyence BZ-X810. (**F**) Representative images and quantification of the 3D invasion assay performed using DLD-1 LIN28B^hi^ cells treated with the vehicle or MOR20350. Scale bar = 500 μm. (**G**) Representative images of RFP expressed by DLD-1 LIN28B^hi^ tumors in the liver. Images were taken using Keyence BZ-X810. Scale bar = 500 μm. Data in graphs **A**–**F** are represented as means ± SEM and were analyzed by 1-way ANOVA followed by Tukey’s multiple-comparison test. Data in **G** were analyzed by Fisher’s exact test. *P < 0.05, ***P < 0.001.

To evaluate the potential of NOTCH3 as a therapeutic target, we used an anti-NOTCH3 antibody MOR20350 (43) to study the effects of NOTCH3 inhibition in vivo and in vitro. qRT-PCR analysis showed no significant difference in *NOTCH3* mRNA expression between MOR20350- and vehicle-treated LIN28B^hi^ DLD-1 and LoVo cells. However, MOR20350 treatment resulted in a significant decrease in the expression of *HES1* (Figure 7A and Supplemental Figure 8A). Cell migration (Figure 7, B and C, and Supplemental Figure 8, B and C), cell aggregation (Figure 7D and Supplemental Figure 8D), and the propensity of the cells to invade through the ECM (Figure 7, E and F, and Supplemental Figure 8, E and F) were significantly decreased in MOR20350-treated LIN28B^hi^ cells when compared with the vehicle-treated Ctrl group. Intraperitoneal injection of the vehicle or MOR20350 after the portal vein injection of LIN28B^hi^ CRC cells resulted in the inhibition of liver metastases’ formation (0/8, 0%) when compared with the vehicle-treated mice (4/7, 57.1%) (Figure 7G and Supplemental Figure 8G). These results further support the contribution of LIN28B/CLDN1/NOTCH3 axis in cell aggregation, cell migration, and collective cell invasion and identify NOTCH3 as a promising pharmacologic target for the treatment of metastatic colon cancer.

### LIN28B/CLDN1/NOTCH3 axis positively correlates with metastatic progression of human colorectal tumors.

**Figure 8:**
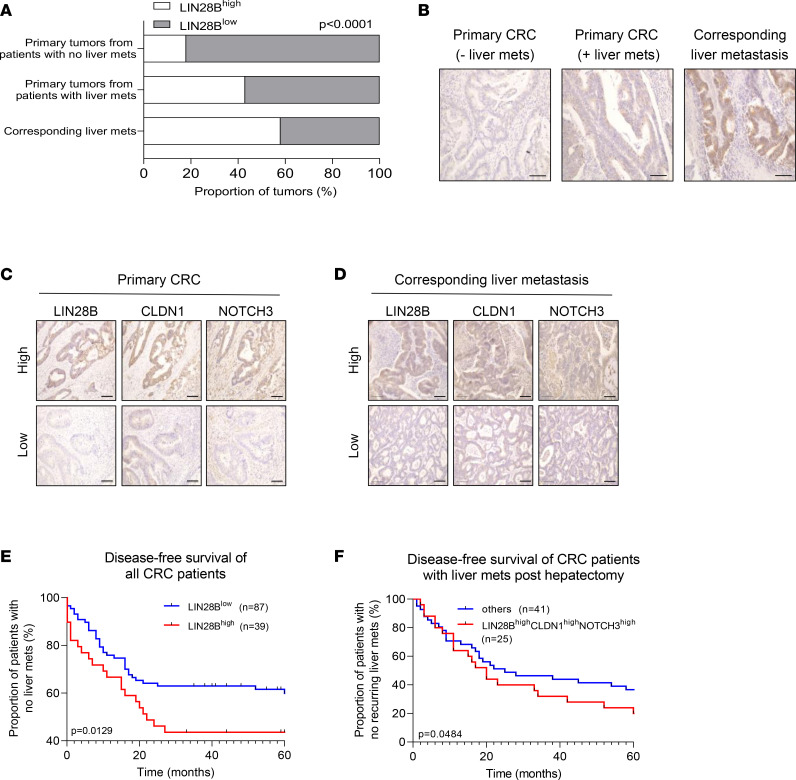
LIN28B/CLDN1/NOTCH3 axis positively correlates with metastatic progression of human colorectal tumors. (**A**) Primary colonic tumors and liver metastases were collected from patients with CRC. Upon IHC staining, tumors were quantified based on their high or low expression. Data expressed as a proportion of all tumors (%). (**B**) Representative images of tumors stained with an anti-LIN28B antibody. Scale bar = 100 μm. (**C**) Representative images of primary colorectal tumors stained with LIN28B, CLDN1, and NOTCH3. Scale bar = 100 μm. (**D**) Representative images of corresponding liver metastases stained with LIN28B, CLDN1, and NOTCH3. Scale bar = 100 μm. (**E**) Graph depicting disease-free survival of all patients with CRC. For all patients, frequency with which they developed liver metastases was tracked over 5 years. Data expressed as the proportion of patients with CRC who did not have metastatic liver tumor (*y* axis) at the time of analyses (*x* axis). (**F**) Graph depicting disease-free survival of patients with CRC who had undergone hepatectomy due to liver metastases. For all patients, frequency with which they developed a new liver tumor was tracked over 5 years. Data expressed as the proportion of patients with CRC who did not develop another metastatic liver tumor (*y* axis) at the time of analyses (*x* axis). “Others” group (blue line) includes patients with tumors that do not have high expressions of LIN28B, CLDN1, and NOTCH3. Data in graph in **A** were analyzed by χ^2^ test. Data in **E** and **F** were analyzed by log-rank test.

To translate our findings into CRC cases in humans, we quantified the expression of LIN28B in tumor tissues obtained from patients with CRC. Of 126 CRC cases observed, 60 cases did not develop liver metastases whereas 66 cases developed liver metastases and required partial hepatectomy (part of standard of care). Tumor samples were divided into (i) primary colorectal tumors from patients who did not develop liver metastases, (ii) primary colorectal tumors from patients who developed liver metastases, and (iii) corresponding liver metastases from the same patients as group ii. Intriguingly, 42% of primary tumors from patients who developed liver metastases and 58% of the corresponding liver metastases had high expression of LIN28B, whereas only 19% of primary tumors from patients who did not have liver metastases had high LIN28B expression (Figure 8, A and B). These data suggest that LIN28B expression may be upregulated in the CRC metastatic cascade.

We next stained for CLDN1 and NOTCH3 in the primary tumors and matched liver metastases collected from patients with CRC. We found 74% of LIN28B^hi^ primary tumors also had high expression of CLDN1, providing evidence for a positive correlation between LIN28B and CLDN1 in primary tumors (Figure 8C and Supplemental Table 1); 48% of LIN28B^hi^ primary tumors also had high expression of NOTCH3 (Figure 8C and Supplemental Table 1). More relevant to the investigation of the metastatic cascade, however, 79% and 63% of LIN28B^hi^ liver metastases had increased expression of CLDN1 and NOTCH3, respectively (Figure 8D and Supplemental Table 1). These data suggest a positive correlation between LIN28B, CLDN1, and NOTCH3 in metastatic human CRC tissues.

To correlate LIN28B expression in primary colorectal tumors with the likelihood of developing liver metastases, we quantified the proportion of patients with CRC who developed liver metastases over 5 years. LIN28B expression in primary tumors was associated with patients with lower relapse-free survival, suggesting that patients with LIN28B^hi^ primary tumors are more likely to develop liver metastases in 5 years when compared with patients with LIN28B^lo^ primary tumors (Figure 8E). We also measured the expression of LIN28B, CLDN1, and NOTCH3 in primary tumors collected from patients with metastatic CRC posthepatectomy. The same patients were followed over 5 years to track their recurrence of liver metastases. Interestingly, a greater proportion of patients with tumors that expressed high levels of LIN28B, CLDN1, and NOTCH3 developed more liver metastases following a hepatectomy procedure when compared with all other patients (Figure 8F). Together, our data indicate a positive correlation between LIN28B/CLDN1/NOTCH3 and CRC metastasis to the liver.

## Discussion

The RBP LIN28B is associated with tumor development, invasion, and poor prognosis in various types of cancers, such as esophageal, colon, ovarian, prostate, and breast cancers (7–9, 12, 44, 45). We and others have shown that LIN28B expression is associated with metastatic behavior of cancer cells in murine xenograft models (9, 46, 47). More specific to CRC, LIN28B expression is associated with colorectal tumorigenesis, tumor growth, cell migration, and tumor recurrence (48, 49). LIN28 functions as a negative regulator of *let-7*, a family of miRNAs known to regulate the expression of a number of genes involved in development and cellular proliferation. Due to the role of *let-7* as a tumor suppressor, *let-7*–dependent effects of LIN28B in cancer are currently an area of active research (13). For example, a recent study published by Qi et al. revealed a mechanism by which LIN28B promotes lung metastasis of breast cancer by building an immune-suppressive premetastatic niche, and this process may be dependent on the release of exosomes that express low levels of *let-7* (50). Interestingly, recent studies have suggested that LIN28B may also regulate cancer progression in a manner independent of *let-7* (9, 27, 51). In the present study, we investigated the mechanism by which LIN28B promotes collective cell invasion, migration, and metastasis of CRC cells. We provide the first evidence to our knowledge for the direct binding of LIN28B with *CLDN1* mRNA. Upon stabilization by LIN28B, CLDN1 induces expression of NOTCH3 to promote cell aggregation and collective cell invasion. Activation of this LIN28B/CLDN1/NOTCH3 axis mediates the metastatic cascade of CRC. In line with the established importance of cell cluster formation in cancer metastasis to secondary sites (52, 53), our data suggest that LIN28B promotes cancer cell invasion by maintaining tight junctions of colorectal epithelial cells, allowing for the formation of cellular aggregates that likely circulate as part of the metastatic cascade.

The tight junction complex is one of several clusters of proteins that reside between epithelial cells to bind the cells together and form a physiological barrier. The transmembrane protein CLDN1 is integral to the structure and function of tight junctions expressed by colonic epithelial cells (14–16). Despite being the most studied claudin in cancers to date, the role of CLDN1 in cancer progression is controversial and has been a subject of discussion in cancer research. The role of CLDN1 as a tumor suppressor has been demonstrated in prostate cancer (54), lung adenocarcinoma (55), and estrogen receptor–positive subtypes of breast cancers (56). Loss of CLDN1 has also been shown to be a strong predictor of disease recurrence and poor patient survival in CRC (57). By contrast, a tumor-promoting role of CLDN1 has also been highlighted. High CLDN1 expression is found in thyroid carcinomas (58, 59) and correlates with shorter overall survival of patients with gastric (60) and ovarian (61) cancers. Additionally, TNF-α–mediated CLDN1 expression is associated with increased proliferation of pancreatic cancer cells (62). In CRC, CLDN1 overexpression has been associated with invasion and metastasis through the regulation of E-CADHERIN, matrix metalloproteinase-9, β-catenin signaling, and the PI3K/Akt pathway (63), but these studies have been descriptive and nonmechanistic.

In our study, we demonstrate that suppression of CLDN1 inhibited LIN28B-induced invasion and migration of CRC cells. Additionally, we found that suppression of CLDN1 inhibited cell aggregation. With the establishment that tumor cells circulate in clusters (64), maintenance of this clustered state by CLDN1-mediated cell adhesion may be key to acquiring the metastatic properties of CRC cells. In line with this, our findings support the notion that CLDN1 upregulation by LIN28B contributes to cell aggregation and collective cell invasion of CRC cells. Another proposed mechanism by which CLDN1 regulates cancer metastasis is by its functional role in cellular transformation. CLDN1 has been suggested to promote epithelial-mesenchymal transition (EMT), allowing for migration and metastasis in CRC (19, 65). CLDN1 may be the link between LIN28B and EMT and may explain how LIN28B induces EMT during CRC metastasis, a phenomenon that we have described previously (37).

Our investigation into the downstream effectors of CLDN1 revealed NOTCH3 as an important regulator of LIN28B/CLDN1-mediated CRC metastasis. Both downregulation of NOTCH3 and pharmacologic inhibition of the Notch signaling pathway led to decreased cellular aggregation, invasion, and metastasis of CRC cells. The Notch signaling pathway has emerged as a critical regulator of various types of cancer, leading to the generation of Notch pathway inhibitors such as γ-secretase inhibitors (66) and monoclonal antibodies (67, 68). Despite having promising indications in hematological and solid tumors, one of the limitations of Notch signaling inhibition is intestinal toxicity, leading to the reliance on intermittent dosing of the inhibitors (69, 70). Targeting individual Notch1, Notch2, and Notch3 paralogs may bypass the human toxicities of pan-Notch inhibition. Our work has identified NOTCH3 as an important regulator of cellular aggregation, invasion, migration, and liver metastasis formation. Consistent with the findings by Varga et al. (71), inhibition of NOTCH3 led to reduced CRC metastasis in mice, offering a rationale for specific targeting of NOTCH3 to manage CRC metastasis.

Our analyses of tumor samples collected from patients with CRC revealed a positive correlation between LIN28B, CLDN1, and NOTCH3 expression and CRC metastasis to the liver. Furthermore, the likelihood of CLDN1 and NOTCH3 expression seems to be dependent upon LIN28B expression, further supporting our discovery that CLDN1 and NOTCH3 function downstream of LIN28B. Our clinical data suggest that LIN28B, CLDN1, and NOTCH3 may be used as potential prognostic markers of CRC metastasis to the liver. Additionally, LIN28B expression negatively correlated with disease-free survival status of the patients, implicating a greater likelihood of patients with high LIN28B-expressing tumors developing metastases.

Taken together, our results indicate that LIN28B promotes cell aggregation, invasion, and liver metastasis in CRC through posttranscriptional induction of CLDN1 and upregulation of NOTCH3. Development of new therapies that target LIN28B/CLDN1/NOTCH3 axis may provide an effective strategy for inhibiting CRC metastasis to the liver.

## Methods

### Cell culture.

DLD-1 (RRID: CVCL_0248), Caco-2 (RRID: CVCL_0025) and LoVo (RRID: CVCL_0399) cells were purchased from the American Type Culture Collection. All cell lines were authenticated by short tandem repeat profiling analysis. The cells were cultured in DMEM supplemented with 10% FBS (GE Healthcare Life Sciences, now Cytiva), with penicillin-streptomycin (100 IU/mL, Thermo Fisher Scientific), at 37°C in a humidified incubator with 5% carbon dioxide (CO_2_). Experiments were performed within at most 15 passages after thawing. The absence of mycoplasma was confirmed every 2 months using MycoAlert Mycoplasm Detection Kit (Lonza, LT07-118).

### Establishment of LIN28B overexpression cell lines.

LoVo and DLD-1 cells with stable LIN28B expression were established using MSCV-PIG vector plasmids provided by Joshua Mendell (Department of Molecular Biology, University of Texas Southwestern Medical Center, Dallas, Texas, USA) as described previously (9). Viral particles were generated in Phoenix A cells (ATCC). The transduced CRC cells were selected by 2 μg/mL puromycin for DLD-1 and LoVo cells and 5 μg/mL for Caco-2 cells.

### Knockdown of LIN28B, CLDN1, or NOTCH3 expression in colon cancer cells.

BII-mirT3G-Puro vector was generated and used for LIN28B knockdown in Caco-2 cells as described previously (12). For knockdown of CLDN1 and NOTCH3 expression, shRNAs designed by Genecopoeia were used. After making lentiviral particles in HEK293T (Thermo Fisher Scientific), CRC cells with LIN28B^hi^ expression (LoVo LIN28B^hi^, Caco-2 Ctrl, and DLD-1 LIN28B^hi^) were transduced using 8 μg/mL of polybrene (MilliporeSigma) and subjected to 30 minutes’ spinning at 1,200*g*. The transduced cells were selected by hygromycin (MilliporeSigma). shRNA particles used are shown in Supplemental Table 2.

### qRT-PCR.

Total RNA was isolated using the GeneJET RNA Purification Kit (Thermo Fisher Scientific, K0731) according to the manufacturer’s protocol. To synthesize cDNA, 1 μg of total RNA was reverse-transcribed using 25 units of MultiScribe Reverse Transcriptase (Invitrogen, 4311235) and 0.5 μg of oligo-dT primer (Invitrogen, 18418012). qRT-PCR was performed on the StepOnePlus Real-Time PCR System (Applied Biosystems, 4376600) using 1 μL of cDNA, 0.5 μM of primers, and 5 μL of Power SYBR Green PCR Master Mix (Applied Biosystems, 4367659) per 10 μL reaction. Primer sequences are listed in Supplemental Table 3.

### Western blot.

Protein samples were extracted using Cell Lysis Buffer (Cell Signaling Technology, 9803) containing 1 mM phenylmethylsulfonyl fluoride and a protease inhibitor cocktail (MilliporeSigma, P8340). Protein concentration was quantified by the Bradford method using the Protein Assay Dye Reagent Concentrate (Bio-Rad, 5000006). Electrophoresis was performed with NuPAGE 4%–12% Bis-Tris gels and MOPS SDS Running Buffer (Invitrogen, NP000102). Proteins were transferred to a polyvinylidene difluoride membrane (Immobilon- FL, MilliporeSigma, IPFL00010) using NuPAGE Transfer Buffer (Invitrogen, NP0006). Primary antibodies used for immunoblotting are listed in Supplemental Table 4. IRDye secondary antibodies (LI-COR, 926-68070, 926-32213) were used before measurement. All protein measurements were normalized to GAPDH used as an endogenous control.

### mRNA stability assay.

A total of 5.0 × 10^5^ cells were seeded in a 6-well culture plate. After 24 hours, culture medium was changed to medium containing 5–15 μg/mL actinomycin D (MilliporeSigma, A1410) to inhibit transcription. After incubation for up to 4 hours, total RNA was extracted. qRT-PCR was performed as described above.

### Cell aggregation assay.

The ability of cell aggregation was assessed by measuring the number of colonies after seeding single cells. For LoVo and DLD-1 cells, 1.0 × 10^4^ cells were seeded into 96-well, flat-bottom, ultra-low-attachment plates (Corning, 3474). For Caco-2 cells, 5.0 × 10^3^ cells were seeded. After incubation for 24 hours, the number of cell aggregates over 2,000 μm^2^ was counted by Keyence BZ-X810.

### Scratch wound-healing assay.

Confluent monolayer cells in 6-well plates were scratched using a 200 μL pipette tip. Images of wound closure were captured at 0, 24, and 48 hours after the scratch. The wounded area was measured using ImageJ software as previously described (72). The wound-healing rate was normalized to the area of the initial scratch wound.

### Transwell migration and invasion assay.

Membrane pores (8 μm, 353097, Corning) for 24-well plates were used to measure cell migration. A total of 1.0 × 10^5^ (Caco-2) or 5.0 × 10^4^ (DLD-1, LoVo) cells in 100 μL serum-free medium were added to the upper chamber, and 100 μL of 10% FBS medium was added in the lower chamber. After cells were incubated at 37°C for 24 hours, nonmigratory cells in the top chamber were removed by cotton swabs. To analyze invasiveness, the Transwell inserts were first coated with Matrigel (Corning, 354480). Migratory or invasive cells at the bottom of the membrane were fixed in 70% ethanol and stained by 0.2% crystal violet. The number of migratory or invasive cells was counted in 3 fields by microscopy (Keyence, BZ-X810).

### Spheroid 3D invasion assay.

A total of 1.0 × 10^3^ cells in 100 μL of culture medium were seeded into 96-well, round-bottom, ultra-low-attachment plates (Corning, 7007) and incubated for 3 days to form spheroid tumors. After formation of tumor spheroids, 70 μL of culture medium was carefully removed. To optimize the ECM conditions, the same amount of Matrigel (Corning, 354480) and Rat Tail Collagen type 1 (Corning, 354236) was mixed. A total of 70 μL of the mixed solution was added to each well and incubated at 37°C for 30 minutes to form the gel. After 30 minutes, 100 μL of warm culture medium was added. The growth of a tumor spheroid was imaged at days 1, 2, 3, and 7, and the area of tumor cells was calculated using ImageJ software (72).

### RIP.

The samples were extracted from CRC cells with LIN28B^hi^ expression (LoVo LIN28B^hi^, Caco-2 Ctrl, DLD-1 LIN28B^hi^). LIN28B ribonucleoprotein particles were immunoprecipitated using SureBeads Protein G Magnetic Beads (Bio-Rad, 161-4023) and RIP-Assay Kit (MBL, RN1001) according to the manufacturer’s protocol. An equal amount of normal rabbit IgG was used as a negative control. RNA in the RIP products was analyzed by qRT-PCR. CLDN1 expression levels in the LIN28B antibody immunoprecipitates were compared with those in the control rabbit IgG antibody immunoprecipitates.

### Immunocytochemistry.

A total of 5.0 × 10^4^ cells were seeded on round coverslips in 24-well plates. After 2 days, cells were fixed for 5 minutes at –20°C in 100% methanol. Immunostaining was performed according to Thermo Fisher Scientific’s protocol. Primary antibodies used for immunofluorescence are listed in Supplemental Table 4. Cy3-conjugated secondary antibody was obtained from Jackson Immunoresearch Laboratories (1:600, 715-165-150). Coverslips were mounted using Vectashield Antifade Mounting Medium with 4′,6-diamidino-2-phenylindole (Vector Laboratories, H-1200) and imaged by fluorescence microscopy (Keyence, BZ-X810).

### Animal experiments.

Female athymic nude mice (CrTac NCr-Foxn1^nu^) between 6 and 8 weeks of age were obtained from Taconic Biosciences (model NCRNU). *Vil^Cre^ Lin28b*-low or -high mice were generated by our lab (12). *Vil^Cre^* mice were purchased from The Jackson Laboratory. All mice were acclimated for 1 week before use. Animal experiments were conducted in accordance with a protocol approved by the Columbia University Institutional Animal Care and Use Committee.

### Portal vein injection.

The nude mice were anesthetized with isoflurane. A total of 2.0 × 10^6^ cells were suspended in 100 μL of PBS and were injected into the portal vein of nude mice during open laparotomy with 28G needle. After 4 weeks, the mice were sacrificed by CO_2_ inhalation and the livers were removed. The resected liver tissues were fixed in 10% neutral buffered formalin for a day and placed in 70% ethanol prior to being embedded in paraffin. Paraffin sections of liver tissues were generated every 800 μm thickness and stained with hematoxylin and eosin.

### In vivo and in vitro DAPT and MOR20350 treatments.

Cells were incubated with 10 μM or 20 μM DAPT dissolved in 0.1% dimethyl sulfoxide (DMSO) for 48 hours. Equal volume of DMSO was used as a negative control. A total of 10 μg/mL of MOR20350 (Novartis) (43) was dissolved in the buffer prior to incubation with the cells for 48 hours. For in vivo experiments using DAPT, mice received 100 μL of 1 mg/kg of DAPT every other day by intraperitoneal injections for 4 weeks. For in vivo experiments using MOR20350, mice received 100 μL of 20 mg/kg of MOR20350 dissolved in dilution buffer every other day by intravenous injections for 4 weeks. Mice in the control group were given either 100 μL DMSO or IgG control antibody.

### Immunohistochemistry.

Antigen retrieval was performed by heating slides in 10 mM citric acid buffer using a pressure cooker. The nonspecific staining was blocked by 3% hydrogen peroxide, 0.002% avidin, 0.002% biotin, and StartingBlock Blocking Buffer (Thermo Fisher Scientific, 37539). Primary antibodies used for immunohistochemistry are listed in Supplemental Table 4. Biotinylated secondary antibody was obtained from Vector Laboratories (1:200, BA-1000). Signals were detected using ABC-HRP kit (Vector Laboratories, PK-6100) and DAB substrate kit (Vector Laboratories, SK-4100).

### Immunohistochemistry of human tissues.

Immunohistochemical staining of LIN28B/CLDN1/NOTCH3 was performed following standard protocols. Briefly, paraffin-embedded tissue blocks were cut into 4 μm–thick sections. The tissue sections were incubated with anti-CLDN1 (catalog: 71-7800, Invitrogen; dilution 1:1,000), and anti-NOTCH3 (catalog ab23426, Abcam; 1:100) overnight at 4°C; incubated in Envision+ kits (Dako); and visualized using 0.01% 3,3′-diaminobenzidine. The staining intensities varied from 0 (negative), 1+ (weak), 2+ (moderate), to 3+ (strong). The percentage of cells at each staining intensity level was calculated, and H-score was assigned using the following formula; H-score = (1 × % cells 1+) + (2 × % cells 2+) + (3 × % cells 3+). High expression levels were designated as follows: LIN28B ≥ 120, CLDN1 ≥ 200, NOTCH3 ≥ 100. The score of immunohistochemical staining was evaluated independently by 2 investigators.

### Sample preparation for bulk RNA sequencing.

Single metastatic CRC cells were isolated from metastatic liver tumors after portal vein injection. After mice were euthanized by CO_2_, the liver tissues including CRC metastasis were minced and digested in culture medium containing 200 U/mL collagenase IV (Gibco, 17104019) at 37°C for 30 minutes. The undigested tissues were removed using a 70 μm cell strainer (Fisherbrand, Thermo Fisher Scientific, 22363547). After centrifugation (300*g*, 4°C, 15 minutes), red blood cells were lysed by adding ACK Lysing Buffer (Gibco, A1049201). The cell pellet was washed by PBS containing 0.04% BSA and resuspended by FACS buffer containing 0.5% BSA, 1 mM EDTA, and 0.1% sodium azide in PBS. The metastatic CRC cells were sorted by a flow cytometer in the presence of mCherry fluorescence (BD Biosciences Influx Cell Sorter). Normal mouse liver tissues were used as a negative control for gating. Total RNAs were extracted using RNeasy Plus Micro Kit (QIAGEN, 74034). The RNA integrity numbers (RINs) were measured by RNA 6000 Pico Kit and 2100 Bioanalyzer (Agilent). Samples with RIN greater than 7 were used for sequencing. Two biological replicates per sample were prepared.

### Collection of patient samples.

Human CRC and corresponding liver metastasis tissues were obtained from patients who underwent surgical resection at the Department of Frontier Surgery (for primary CRCs) or General Surgery (for liver metastases), Chiba University Hospital, Japan, from 2005 to 2014. All patients were histologically diagnosed with primary colorectal adenocarcinoma. The study protocol has been approved by the Ethics Committees of Chiba University, and written informed consent was obtained from each patient before surgery.

### Statistics.

All analyses and experiments were repeated at least 3 independent times (minimum 3 technical replicates and 3 biological replicates). Statistical analyses were performed using 2-tailed Student’s *t* tests when comparing 2 groups or standard ANOVA with Tukey’s multiple-comparison test when comparing 3 or more groups. For animal experiments and microarray data sets, associations between binary categories were analyzed by Pearson’s χ^2^ test or Fisher’s exact test. For analyses using patient samples and data collected from patients, accumulative rates were calculated by using the Kaplan-Meier method, and the significance of difference in survival rate was analyzed by the log-rank test. Fisher’s exact tests were used to compare the distribution of a categorical variable in a group with the distribution in another group with *P* < 0.05 as statistically significant. *P < 0.05, **P < 0.01, ***P < 0.001, ****P < 0.0001. Statistical analysis was performed using GraphPad Prism software version 8.0. All data and analyses were reviewed with our Biostatistics Shared Resource at Columbia University Irving Medical Center.

### Study approval.

Animal experiments were conducted in accordance with a protocol approved by the Columbia University Institutional Animal Care and Use Committee. For collection of patient samples, the study protocol has been approved by the Ethics Committees of Chiba university, and written informed consent was obtained from each patient before surgery.

### Data availability.

The RNA-Seq FASTQ files have been deposited in the Gene Expression Omnibus database of the NCBI, NIH, and are accessible under accession number GSE234513. Values for all data points found in graphs are in the Supporting Data Values file.

## Author contributions

YM, K Sugiura, K Suzuki, AES, NS, and AKR contributed to conceptualization and methodology, data curation, formal analyses, and validation. YM, K Sugiura, K Suzuki, AES, and AKR wrote the manuscript. YM, K Sugiura, K Suzuki, AES, NS, HM, MO, PAS, CJL, and AKR reviewed and edited the manuscript. AKR acquired funding, managed project administration, and supervised the study. The basis for the designation of co–first authors was equal contribution to experimental design, generation of data, and data analysis. Each of the co–first authors contributed to the design of the experiments through open and transparent discussions. Each of the co–first authors developed the manuscript through shared writing assignments. Having provided the rationale for the 4 co–first authors, we now pivot to the specific order. This was based upon the length of engagement of the individual authors with the project.

## Supplementary Material

Supplemental data

Supporting data values

## Version 1 06/15/2023

In-Press Preview

## Version 2 07/24/2023

Electronic publication
