# Supplementary material for: LIN28B promotes cell invasion and colorectal cancer metastasis via CLDN1 and NOTCH3
Source: JCI Insight. 2023 Jul 24;8(14):e167310. doi: 10.1172/jci.insight.167310 (PMC10443801; doi:10.1172/jci.insight.167310)
Supplement: Supplemental data [file jciinsight-8-167310-s128.pdf]

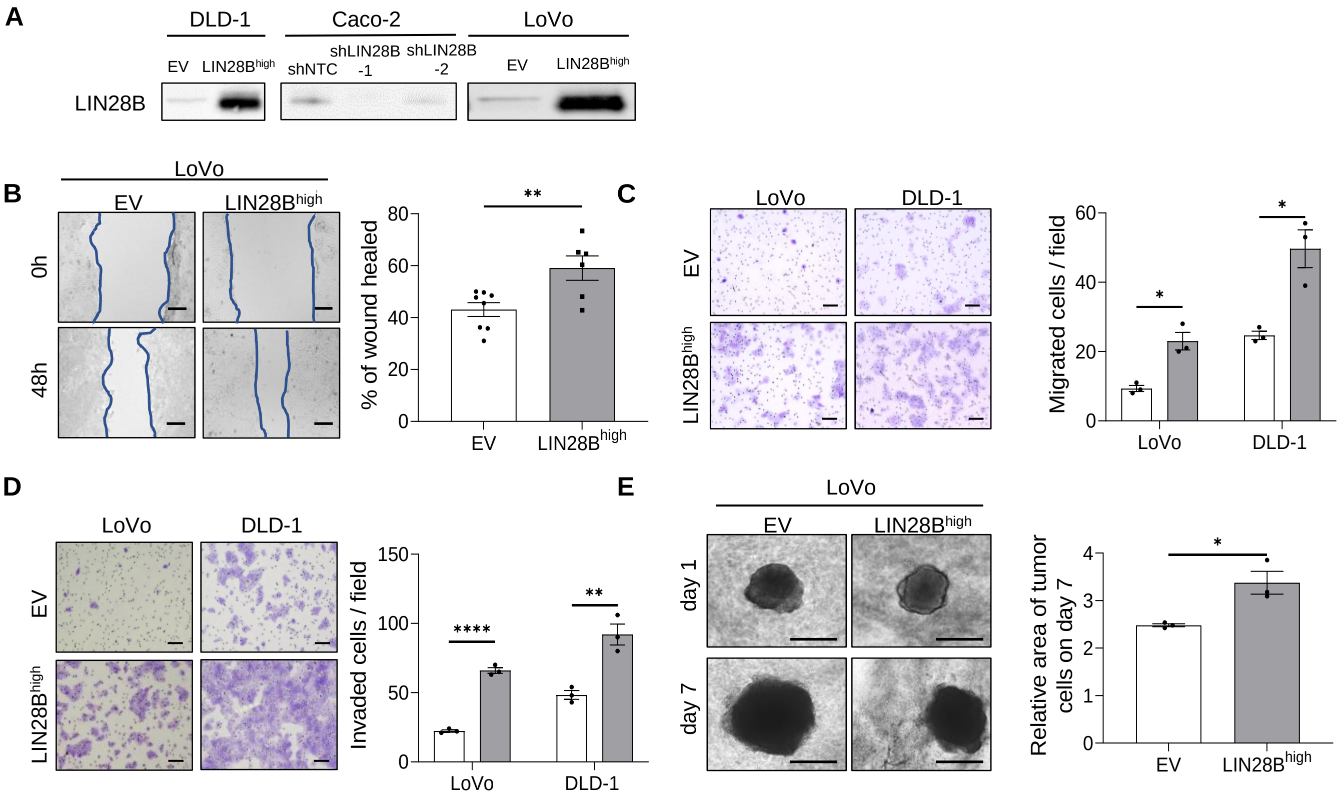

Supplemental Figure S1. LIN28B promotes cell migration and collective cell invasion of CRC cells. (A) Representative images of Western blots to measure LIN28B protein expression in DLD-1, Caco-2, and LoVo cells after overexpressing or knockdown of LIN28B. (B) Wound closure of LoVo with empty vector and LIN28B overexpression. Scale bar = 200 μm. The graph on right panel shows quantification of the wound area measured by ImageJ. Wound healing rate was defined as '(initial wound area - wound area at 24 or 48 hours) / initial wound area'. Data represented as means ± SEM (n=3). (C) 2D transwell migration of LoVo and DLD-1 cells with empty vector and LIN28B overexpression. The graph on right panel shows quantification of the number of migrated cells in fields and is represented as means ± SEM (n=3). (D) 2D transwell invasion of LoVo and DLD-1 cells with empty vector and LIN28B overexpression. The graph on right panel shows the number of invaded cells in fields and is represented as means ± SEM (n=3). (D) 3D invasion of LoVo cells with empty vector and LIN28B overexpression at 1 and 7 days after incubation. Scale bar = 500 μm. The graph on right panel shows tumor area measured by Image J. Data expressed relative to corresponding value on day 1 and is represented as means ± SEM (n=3). For all experiments, *p<0.05, **p<0.01, Student t test.

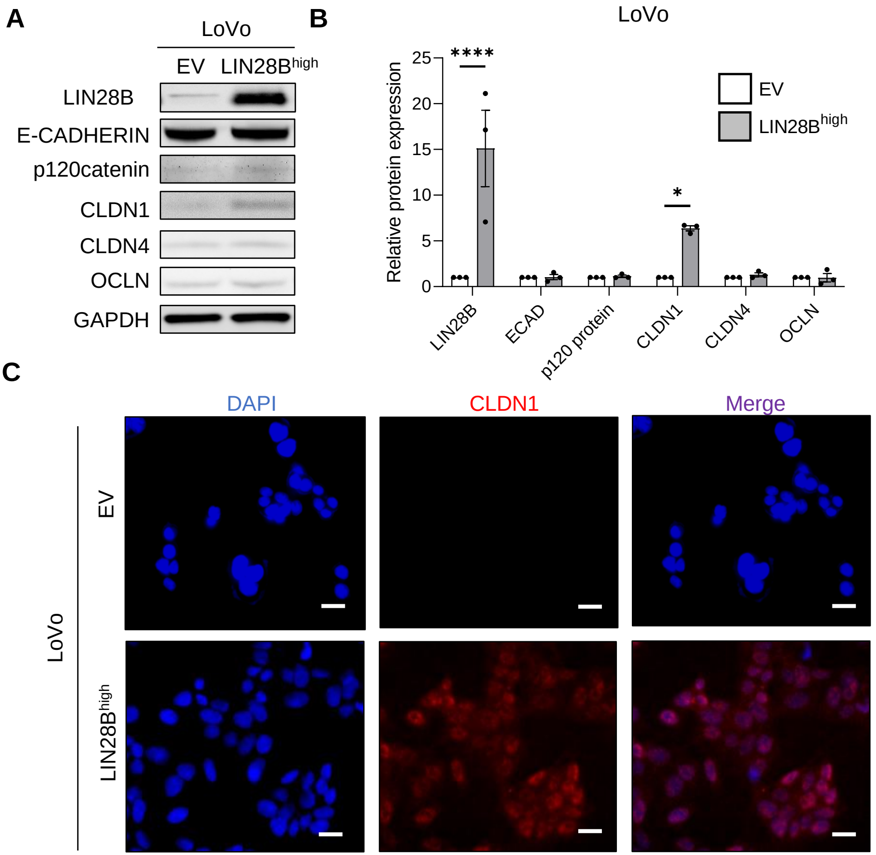

Supplemental Figure S2. LIN28B overexpression induces CLDN1 upregulation in LoVo cells. (A) Western blot analysis of proteins related to adherens and tight junctions in LoVo cells with empty vector and LIN28B overexpression. (B) Quantification of the band densities in (A), normalized to GAPDH endogenous control. Data expressed as relative to the corresponding value of cells with empty vector or control and represented as means ± SEM (n=3). (C) Immunofluorescent staining of CLDN1 (red) in LoVo with empty vector and LIN28B overexpression. Nuclei were stained by DAPI (blue). Scale bar = 100 μm. For all experiments, *p<0.05, **p<0.01, Student t test.

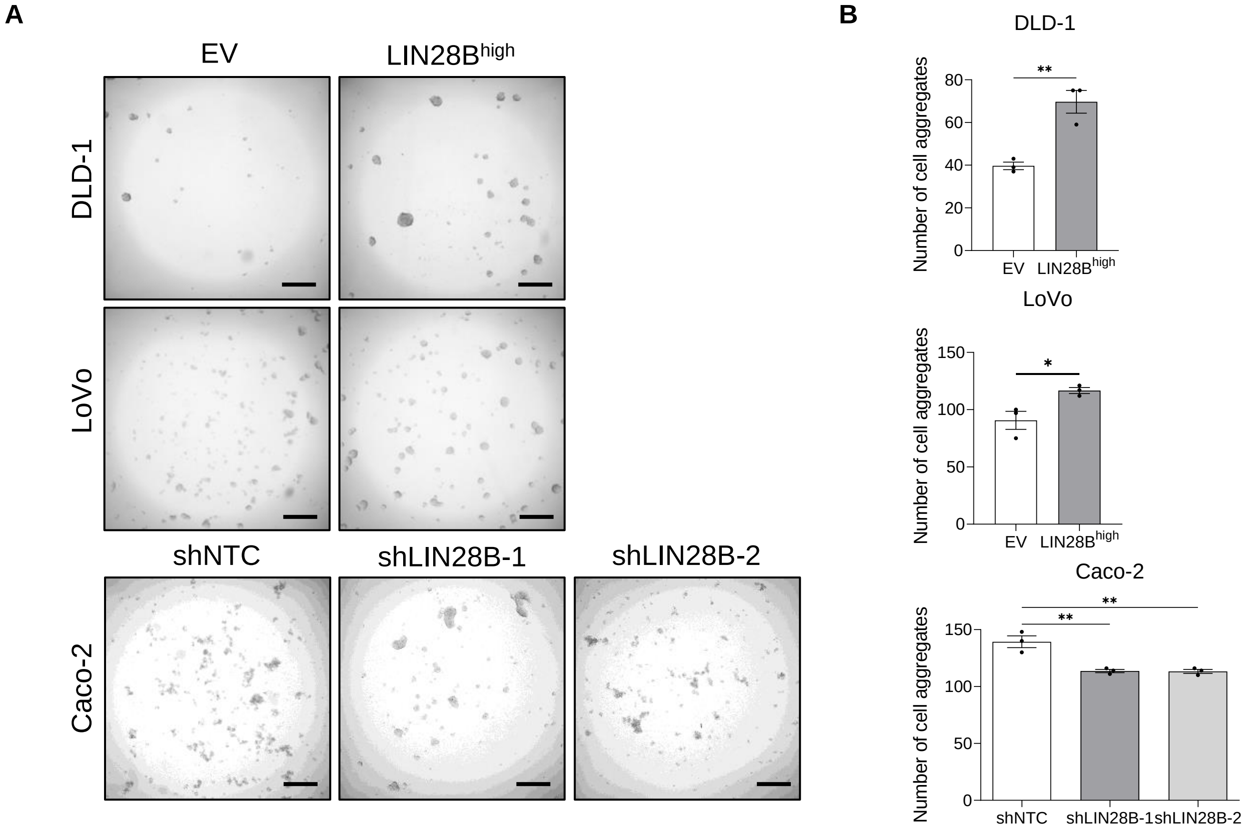

Supplemental Figure S3. LIN28B regulates CRC cell aggregation. (A) Representative images of aggregated DLD-1 and LoVo cells with LIN28B overexpression, and Caco-2 cells with LIN28B knockdown taken after 24-hour incubation in ultra-low attachment plate. Scale bar = 500 μm. (B) Quantification of cell aggregates counted by Keyence BZ-X810 microscope. Data represented as means ± SEM (n=3).

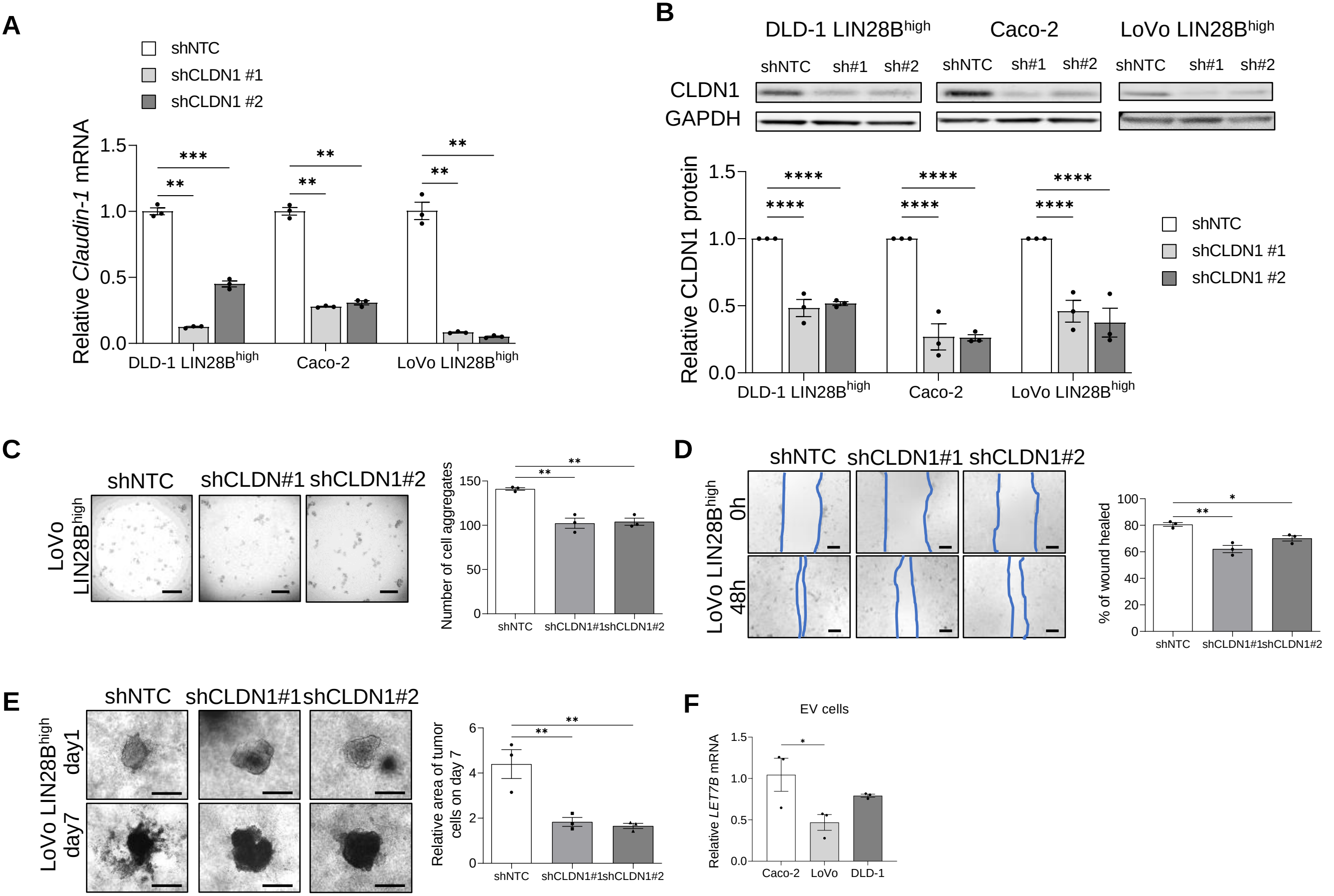

Supplemental Figure S4. Suppression of CLDN1 inhibits collective cell invasion of CRC cells. (A) qRT-PCR analysis of CLDN1 mRNA in colon cancer cells transduced by shNTC, shCLDN1 #1 or shCLDN1 #2. Data expressed relative to corresponding value of control cells and represented as means ± SEM (n=3). (B) Western blot analysis of CLDN1 in CRC cells transduced by shNTC, shCLDN1 #1 or shCLDN1 #2. The panel below shows the band densities normalized to GAPDH. Data expressed relative to the corresponding value of control cells and represented as means ± SEM (n=3). (C) Cell aggregation of LoVo LIN28B^{high} cells transduced by shNTC, shCLDN1 #1 or shCLDN1 #2 after 24-hour incubation in ultra-low attachment plate. Scale bar = 500 μm. The graph on the right panel shows the number of cell aggregates. Data represented as means ± SEM (n=3). (D) Wound closure of LoVo LIN28B^{high} cells transduced by shNTC, shCLDN1 #1 or shCLDN1 #2. Scale bar = 200 μm. The panel below showed the wound healing rate analyzed by using ImageJ. Data represented means ± SEM (n=3). (E) 3D invasion of LoVo LIN28B^{high} cells transduced by shNTC, shCLDN1 #1 or shCLDN1 #2 at 1 and 7 days after incubation. Scale bar = 500 μm. The graph on the right panel shows the tumor area in measured by Image J. Data expressed relative to corresponding value at day 1 and represented as means ± SEM. (F) Relative mRNA expression of LET7B in parental, empty vector colorectal cancer cell lines. Data represented as means ± SEM (n=3).

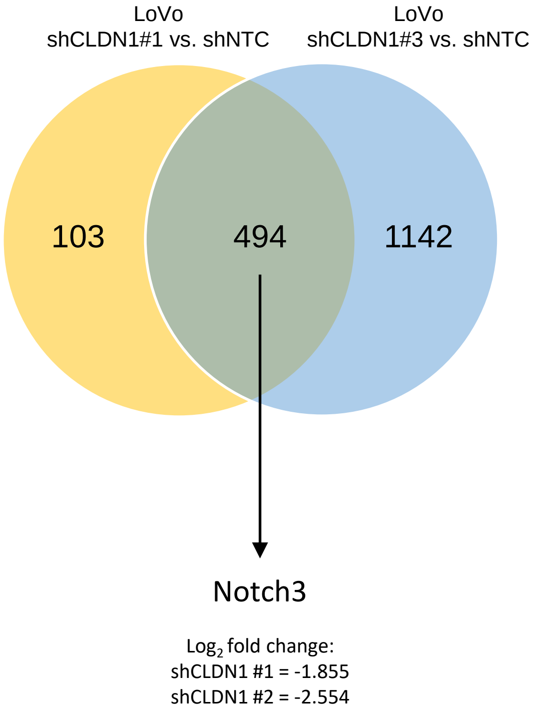

Supplemental Figure S5. NOTCH3 is down-regulated upon CLDN1 knock-down in LIN28Bhigh CRC cells. Venn diagram showing the number of genes that are commonly changed between shCLDN1 #1 and shCLDN1 #2 in LoVo LIN28B^{high} cells.

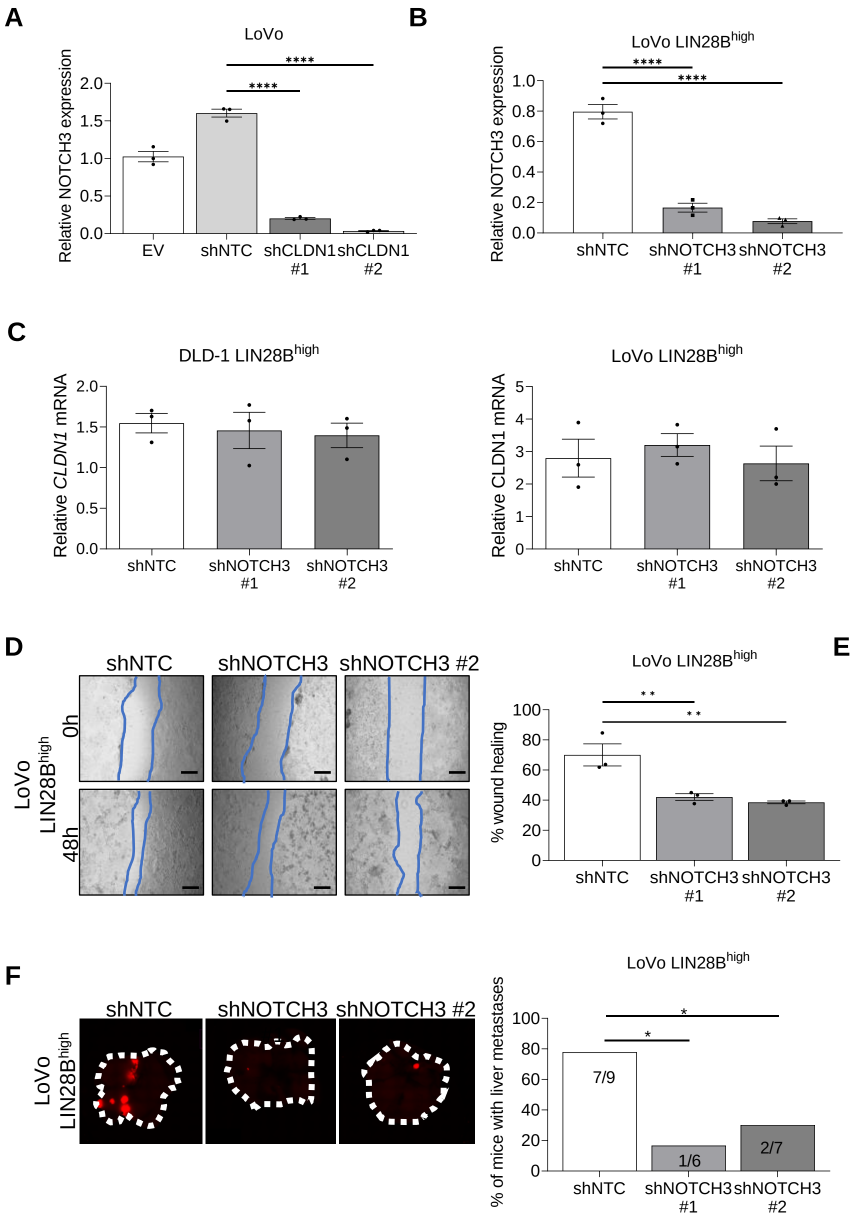

Supplemental Figure S6. Suppression of NOTCH3 inhibits LIN28B-induced cell migration, collective cell invasion, and liver metastasis formation. (A) qRT-PCR analysis of NOTCH3 mRNA in LoVo LIN28B^{high} shNTC, shCLDN1 #1 or shCLDN1 #2. (B) qRT-PCR analysis of NOTCH3 mRNA in LoVo LIN28B^{high} shNTC, shNOTCH3 #1 or shNOTCH3 #2. (C) qRT-PCR analysis of CLDN1 mRNA in DLD-1 and LoVo LIN28B^{high} cells with shNOTCH3 #1 or shNOTCH3 #2. (D) Wound closure of LoVo LIN28B^{high} cells transduced by shNTC, shNOTCH3 #1 or shNOTCH3 #2. Scale bar = 200 µm. Wound healing rate was analyzed by using ImageJ. Data represented as means ± SEM (n=3). (E) Representative images of the aggregation assay in LoVo LIN28B^{high} cells transduced by shNTC, shNOTCH3 #1 or shNOTCH3 #2. The number of cell aggregates were counted by Keyence BZ-X810. Data represented as means ± SEM (n=3). (F) Representative images of liver metastasis, visualized by the RFP expression of LoVo LIN28Bhigh cells and detected by Keyence BZ-X810. The graph on right shows the quantification of the liver metastases expressed as the proportion of mice that have with visible liver tumors.

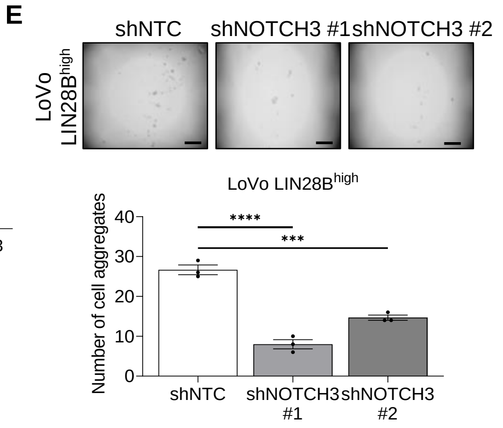

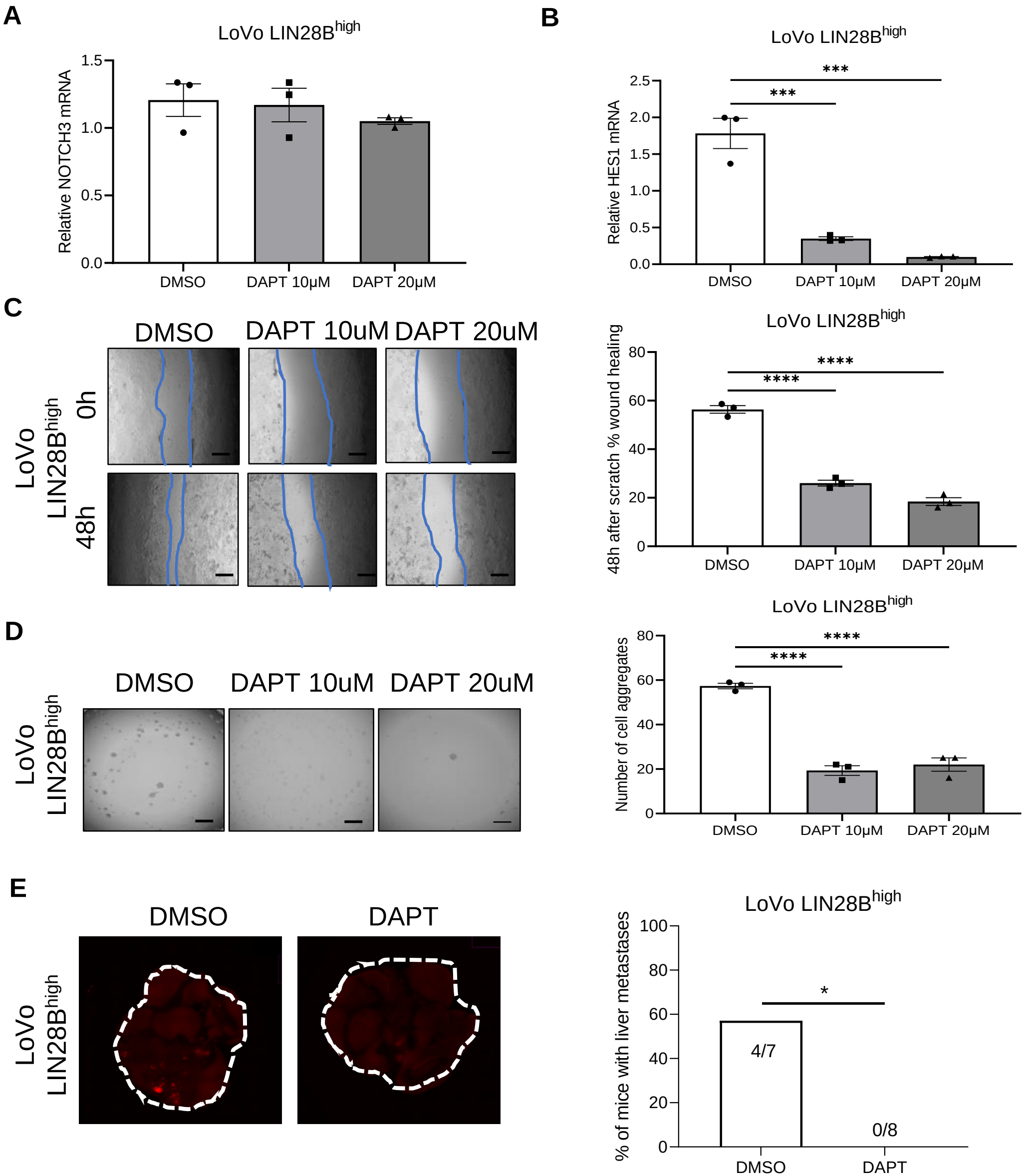
A
LoVo LIN28Bhigh
Relative NOTCH3 mRNA
1.5
1.0
0.5
0.0
DMSO
DAPT 10μM
DAPT 20μM
B
LoVo LIN28Bhigh
Relative HES1 mRNA
2.5
2.0
1.5
1.0
0.5
0.0
***
***
DMSO
DAPT 10μM
DAPT 20μM
C
DMSO
DAPT 10uM
DAPT 20uM
LoVo LIN28Bhigh
0h
48h
LoVo LIN28Bhigh
48h after scratch % wound healing
80
60
40
20
0
****
****
DMSO
DAPT 10μM
DAPT 20μM
D
DMSO
DAPT 10uM
DAPT 20uM
LoVo LIN28Bhigh
LoVo LIN28Bhigh
Number of cell aggregates
80
60
40
20
0
****
****
DMSO
DAPT 10μM
DAPT 20μM
E
DMSO
DAPT
LoVo LIN28Bhigh
LoVo LIN28Bhigh
% of mice with liver metastases
100
80
60
40
20
0
*
4/7
0/8
DMSO
DAPT

Supplemental Figure S7. Inhibition of NOTCH signaling by DAPT inhibits LIN28B-induced collective cell invasion, cell migration, and liver metastasis formation. (A) qRT-PCR analysis of *NOTCH3* mRNA in LoVo LIN28B^{high} cells treated DMSO, 10 µM DAPT or 20 µM DAPT. (B) qRT-PCR analysis of *HES1* mRNA in LoVo LIN28B^{high} cells treated with DMSO, 10 µM DAPT or 20 µM DAPT. (C) Wound closure of LoVo LIN28B^{high} cells treated with DMSO, 10 µM DAPT or 20 µM DAPT. Scale bar = 200 µm. Wound healing rate was analyzed by using ImageJ. Data represented as means ± SEM (n=3). (D) Representative images of cellular aggregation of LoVo LIN28B^{high} cells treated with DMSO, 10 µM DAPT or 20 µM DAPT. The number of cell aggregates were counted by Keyence BZ-X810. Data represented as means ± SEM (n=3). (E) Representative images of liver metastasis, visualized by the RFP expression of LoVo LIN28B^{high} cells and detected by Keyence BZ-X810.

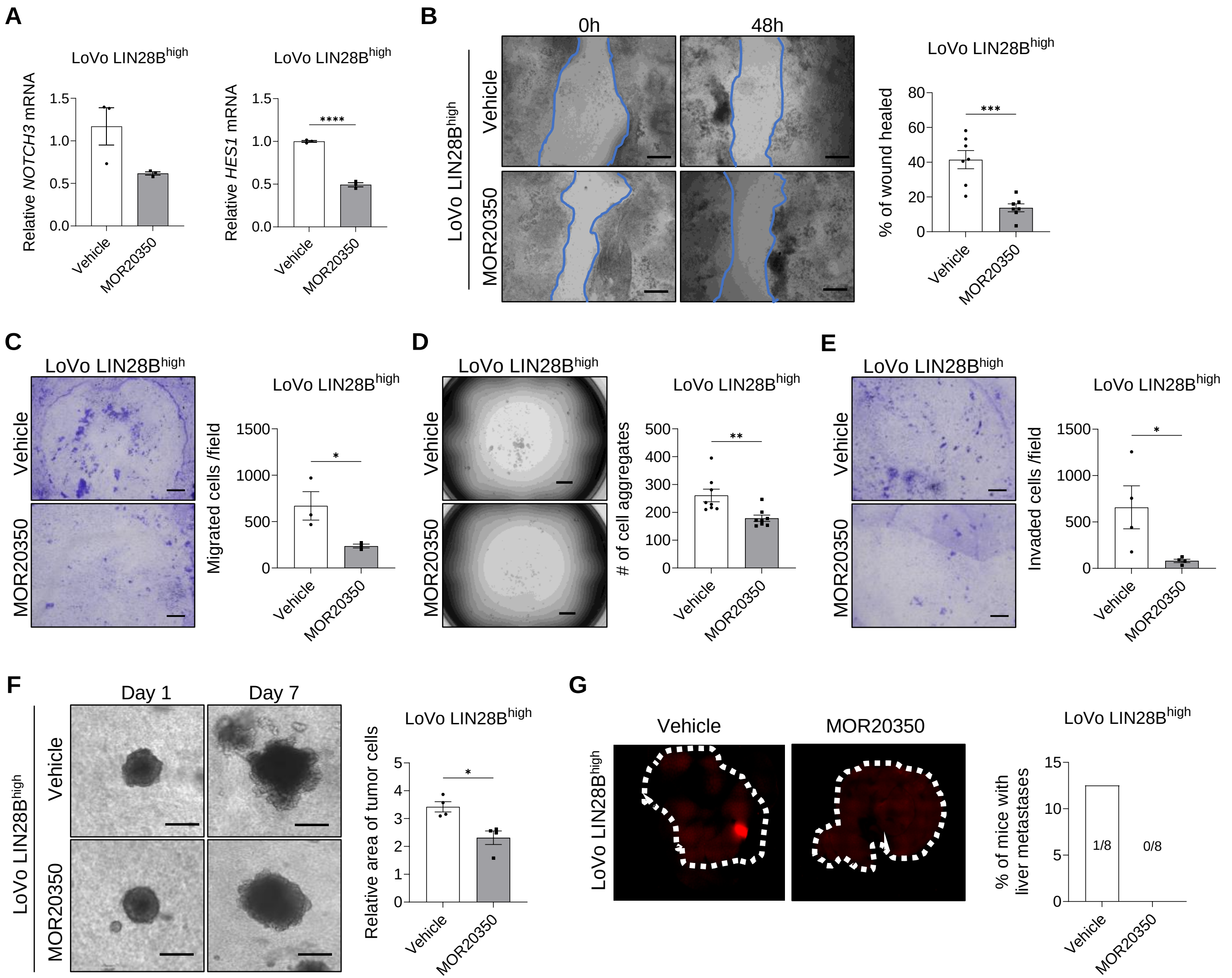

Supplemental Figure S8. Pharmacologic inhibition of Notch3 reduces LIN28B-induced liver metastasis in LoVo cells. (A) qRT-PCR analysis of NOTCH3 and HES1 mRNA in LoVo LIN28B^{high} cells treated with vehicle or 10 μg/mL MOR20350 for 48 hours. (B) Representative images and quantification of the wound closure scratch assay. Scale bar = 200 μm. Area of the wound was measured by using ImageJ. (C) Representative images and quantification of the 3D aggregation assay. The number of cell aggregates were counted using Keyence BZ-X810. (D) Representative images and quantification of 2D invasion assay. Cells that have invaded through the 8 μm pore and the ECM were counted using Keyence BZ-X810. (E) Representative images and quantification of 2D migration assay. Cells that have migrated through the 8 μm pore were counted using Keyence BZ-X810. (F) Representative images and quantification of the 3D invasion assay. Scale bar = 500 μm. (G) Representative images of RFP expressed by LoVo LIN28B^{high} tumors in the liver. Images were taken using Keyence BZ-X810. Data in graphs A-F are represented as means ± SEM and were analyzed by one-way ANOVA followed by Tukey's multiple comparison test. Graph in G was analyzed by Fisher's exact test.

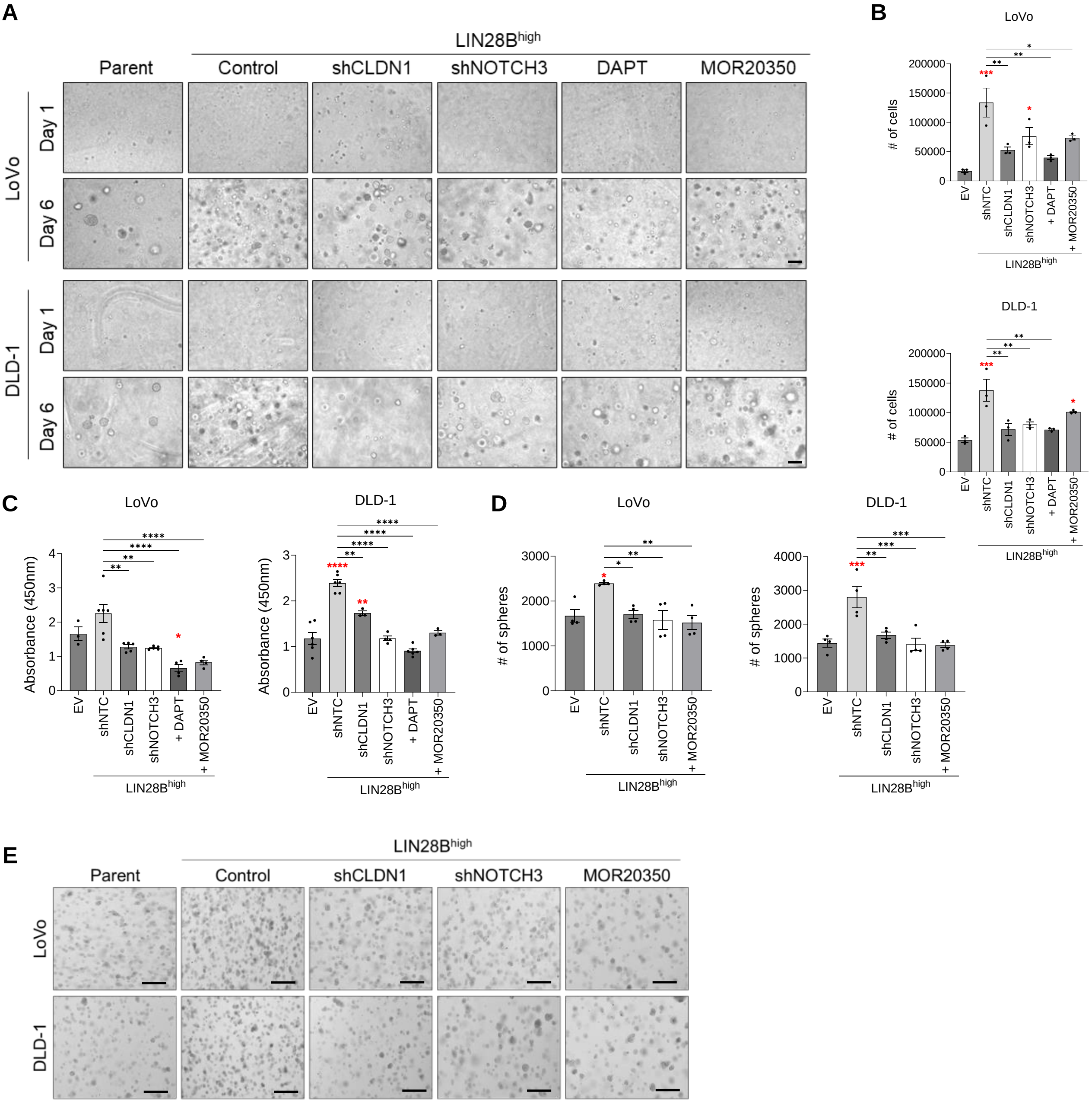
A
LIN28Bhigh
Parent
Control
shCLDN1
shNOTCH3
DAPT
MOR20350
LoVo
Day 1
Day 6
DLD-1
Day 1
Day 6
B
LoVo
# of cells
200000
150000
100000
50000
0
EV
shNTC
shCLDN1
shNOTCH3
+ DAPT
+ MOR20350
LIN28Bhigh
DLD-1
# of cells
C
LoVo
Absorbance (450nm)
DLD-1
D
LoVo
# of spheres
DLD-1
E
LIN28Bhigh
Parent
Control
shCLDN1
shNOTCH3
MOR20350
LoVo
DLD-1

Supplemental Figure S9. The LIN28B-CLDN1-NOTCH3 axis promotes CRC cell proliferation in 2D and 3D cultures. (A) Representative images of colonies formed in soft agar on days 1 and 6 post culture. (B) The number of viable cells in the soft agar colony formation assay were quantified by the 3-(4,5-Dimethylthiazol-2-yl)-2,5-diphenyltetrazolium bromide (MTT) assay on day 6. Data are represented as means ± SEM (n=3). (C) The number of proliferating cells in 2D culture were quantified by WST-1 assay on day 5 post culture. Data represented as means ± SEM (n=3-6). (D) Quantification of the number of spheres counted after 7 days post culture in Matrigel. (E) Representative images of spheres formed after 7 days post culture in Matrigel. Red asterisks above each bar indicate statistical significance compared to the empty vector (EV) control group. Graphs in B and C were analyzed by one-way ANOVA followed by Tukey's multiple comparison test.

Supplemental Table 1. Number of primary colorectal tumors and corresponding liver metastases based on their expression of LIN28B, CLDN1, and NOTCH3.

Primary CRC

	$CLDN1^{high}$	$CLDN1^{low}$
$LIN28B^{high}$	20	7
$LIN28B^{low}$	7	32

$p<0.0001$

	$NOTCH3^{high}$	$NOTCH3^{low}$
$LIN28B^{high}$	13	14
$LIN28B^{low}$	5	34

$P=0.0022$

Corresponding liver metastasis

	$CLDN1^{high}$	$CLDN1^{low}$
$LIN28B^{high}$	30	8
$LIN28B^{low}$	5	23

$p<0.0001$

	$NOTCH3^{high}$	$NOTCH3^{low}$
$LIN28B^{high}$	24	14
$LIN28B^{low}$	4	24

$P=0.0001$

Supplemental Table 2. Target Sequences of shRNA

Name	Catalog Number
shNTC for shLIN28B	CSHCTR001-2-LVRH1GP
shLIN28B #1	HSH101662-LVRH1GP-a
shLIN28B #2	HSH101662-LVRH1GP-c
shNTC for shCLDN1	CSHCTR001-2-LVRU6MH
shCLDN1 #1	HSH088484-LVRU6MH-a
shCLDN1 #2	HSH088484-LVRU6MH-c
shNTC for sh NOTCH3	CSHCTR001-LVRH1GP
shNOTCH3 #1	HSH011876-LVRH1GP-b
shNOTCH3 #2	HSH011876-LVRH1GP-c

Supplemental Table 3. PCR primers

Target gene	Sequence
GAPDH	Forward;5'-TCAAGAAGGTGGTGAAGAAG-3'<br>Reverse; 5'-AAAGGTGGAGGAGTGGGTGT-3'
CLDN1	Forward;5'-CCCTATGACCCCAGTCAATG-3'<br>Reverse;5'-ACCTCCCAGAAGGCAGAGA-3'
NOTCH3	Forward;5'-GCTCAACGGCACTGATCCT-3'<br>Reverse;5'-AGCCCGTGTAAGGCTGATT-3'
HES1	Forward 5′-ACGTGCGAGGGCGTTAATAC-3′<br>Reverse:5′-ATTGATCTGGGTCATGCAGTTG-3′

Supplemental Table 4. List of antibodies

Antibody	Manufacturer and Catalog Number	RRID	Host Species	Clonality	Dilution		
					WB	ICC	IHC
CLDN1	Invitrogen, 37-4900	AB_431448	Mouse	Monoclonal		1:250	
CLDN1	Invitrogen, 71-7800	AB_88416	Rabbit	Polyclonal	1:500		1:100
LIN28B (h)	Cell Signaling Technology, 4196	AB_2135047	Rabbit	Polyclonal	1:1000		
LIN28B (h)	Sigma-Aldrich, HPA061745	AB_2684601	Rabbit	Polyclonal			1:1000
LIN28B (m)	Proteintech, 16178-1-AP	AB_2135051	Rabbit	Polyclonal			1:200
E-CADHERIN	Cell Signaling Technology, 3195	AB_2291471	Rabbit	Monoclonal	1:1000		
p120catenin	BD Transduction, 610134	AB_397537	Mouse	Monoclonal	1:1000		
CLDN4	Invitrogen, 32-9400	AB_2533096	Mouse	Monoclonal	1:500		
OCLN	Invitrogen, 33-1500	AB_2533101	Mouse	Monoclonal	1:1000		
GAPDH	EMD Millipore, MAB374	AB_2107445	Mouse	Monoclonal	1:5000		
GFP	Cell Signaling Technology, 2956	AB_1196615	Rabbit	Monoclonal			1:400
